# Cellular and molecular features of asthma mucus plugs provide clues about their formation and persistence

**DOI:** 10.1172/JCI186889

**Published:** 2025-03-17

**Authors:** Maude A. Liegeois, Aileen Hsieh, May Al-Fouadi, Annabelle R. Charbit, Chen Xi Yang, Tillie-Louise Hackett, John V. Fahy

**Affiliations:** 1 Cardiovascular Research Institute, UCSF, San Francisco, California, USA.; 2 Centre for Heart Lung Innovation, Vancouver, British Columbia, Canada.; 3 Department of Anesthesiology, Pharmacology and Therapeutics, University of British Columbia, Vancouver, British Columbia, Canada.; 4 Division of Pulmonary, Critical Care, Allergy and Sleep Medicine, UCSF, San Francisco, California, USA.

**Keywords:** Immunology, Pulmonology, Asthma

## Abstract

**BACKGROUND:**

Mucus plugs form in acute asthma and persist in chronic disease. Although eosinophils are implicated in mechanisms of mucus pathology, many mechanistic details about mucus plug formation and persistence in asthma are unknown.

**METHODS:**

Using histology and spatial, single-cell proteomics, we characterized mucus-plugged airways from nontransplantable donor lungs of 14 patients with asthma (9 with fatal asthma and 5 with nonfatal asthma) and individuals acting as controls (10 with chronic obstructive pulmonary disease and 14 free of lung disease). Additionally, we used an airway epithelial cell–eosinophil (AEC-eosinophil) coculture model to explore how AEC mucus affects eosinophil degranulation.

**RESULTS:**

Asthma mucus plugs were tethered to airways showing infiltration with innate lymphoid type 2 cells and hyperplasia of smooth muscle cells and MUC5AC-expressing goblet cells. Asthma mucus plugs were infiltrated with immune cells that were mostly dual positive for eosinophil peroxidase (EPX) and neutrophil elastase, suggesting that neutrophils internalize EPX from degranulating eosinophils. Indeed, eosinophils exposed to mucus from IL-13–activated AECs underwent CD11b- and glycan-dependent cytolytic degranulation. Dual-positive granulocytes varied in frequency in mucus plugs. Whereas paucigranulocytic plugs were MUC5AC rich, granulocytic plugs had a mix of MUC5AC, MUC5B, and extracellular DNA traps. Paucigranulocytic plugs occurred more frequently in (acute) fatal asthma and granulocytic plugs predominated in (chronic) nonfatal asthma.

**CONCLUSION:**

Together, our data suggest that mucin-rich mucus plugs in fatal asthma form because of acute goblet cell degranulation in remodeled airways and that granulocytic mucus plugs in chronic asthma persist because of a sustaining niche characterized by epithelial cell–mucin-granulocyte cross-talk.

**FUNDING:**

NIH grants HL080414, HL107202, and AI077439.

## Abstract 2

Variability in the mucin and granulocyte constituents of mucus plugs in fatal and non-fatal asthma suggests that mucus plugs form following acute degranulation of goblet cells and persist in some instances because of a self-sustaining mucin-granulocyte-epithelial cell niche.

## Introduction

Asthma affects over 300 million people globally, and the majority of costs associated with asthma care relate to emergency care and chronic severe disease (1). Asthma is a disease of the airways, and mucus plug pathology is prominent, especially in fatal cases and in chronic severe disease. For example, in cases of fatal asthma, Dunnill described how “the lung showed a striking picture with numerous grey, glistening mucous plugs scattered throughout the air passages” and concluded that “pathologically the outstanding feature of the asthmatic lung lies in the failure of clearance of the bronchial secretions” (2). These and similar pathology findings (3, 4) provide support for widespread airway mucus plugging as a plausible pathophysiologic mechanism of respiratory failure and death from acute fatal asthma. More recently, CT lung imaging has highlighted that mucus plugs are also prevalent in chronic severe asthma and can persist for 3 or more years (5–7). Increased numbers of mucus plugs in patients with asthma are associated with worse airflow obstruction and more frequent exacerbations (5, 6). Currently, there is no approved treatment for mucus plugs in asthma, and the rational design of mucoactive drugs requires better knowledge of the mechanisms that underlie their formation and persistence.

The rapid clinical course of respiratory failure in fatal asthma indicates a mechanism in which mucus plugs form rapidly. Indeed, mice sensitized to aeroallergens quickly form occlusive airway mucus plugs when their airways are challenged with allergen (8). Allergen-mediated mucus plug formation in animal models of asthma involves the release of MUC5AC mucin from airway goblet cells that have undergone metaplasia because of activation of airway epithelial cells by IL-13 (9, 10). However, allergy-related cytokines are not the only regulators of airway mucin expression and goblet cell metaplasia, whose expression and numbers are also influenced by a diverse set of signals downstream of infection (11, 12), environmental pollutants (13, 14), and hypoxia (15). The relativity high prevalence and persistence of mucus plugs in chronic asthma have been a surprising finding in recent studies (5–7), but the mechanisms of mucus plug persistence are not known.

Here, we applied histochemistry, confocal imaging, and spatial, single-cell proteomic imaging to characterize mucus plugs and the airways in which they form in lung tissue from patients with asthma (fatal and nonfatal cases) compared with lung tissue from patients with chronic obstructive pulmonary disease (COPD; disease control) and lung disease–free individuals acting as controls. Our experimental approach was to separately characterize the cellular and molecular features of the airway wall, airway epithelium, and mucus plug compartments, and we focused the protein and cell antibody panel on labeling the mucins, structural cells, and immune cells likely to mediate the acute formation of mucus plugs and their persistence.

## Results

### MUC5AC-rich mucus plugs in asthma are tethered to an epithelium that is buckled to form mucosal folds.

**Figure 1:**
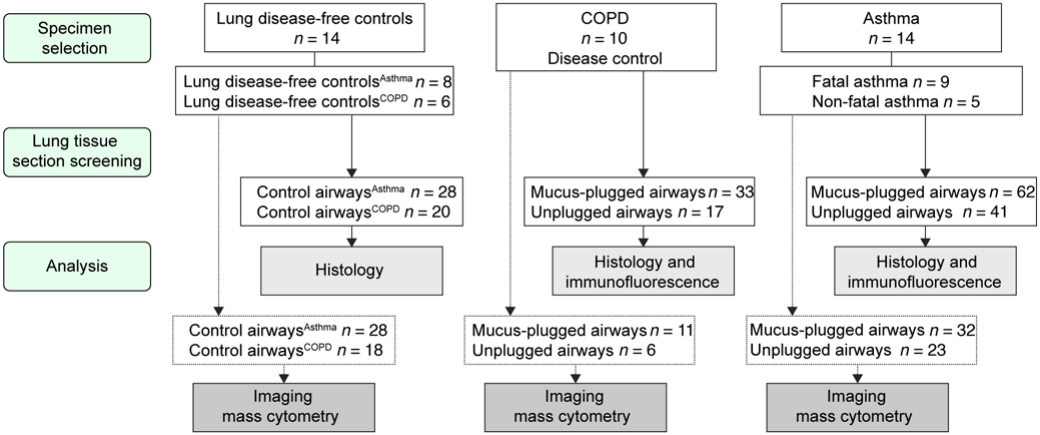
Selection of lung tissues from patients with asthma, patients with COPD, and lung disease–free individuals acting as controls. The flow diagram shows how tissues were selected from the James Hogg Lung Biobank at the University of British Columbia. It further illustrates the screening of lung tissue sections for mucus-plugged and unplugged airways and the number of samples analyzed by histology, immunofluorescence, and imaging mass cytometry.

**Figure 2:**
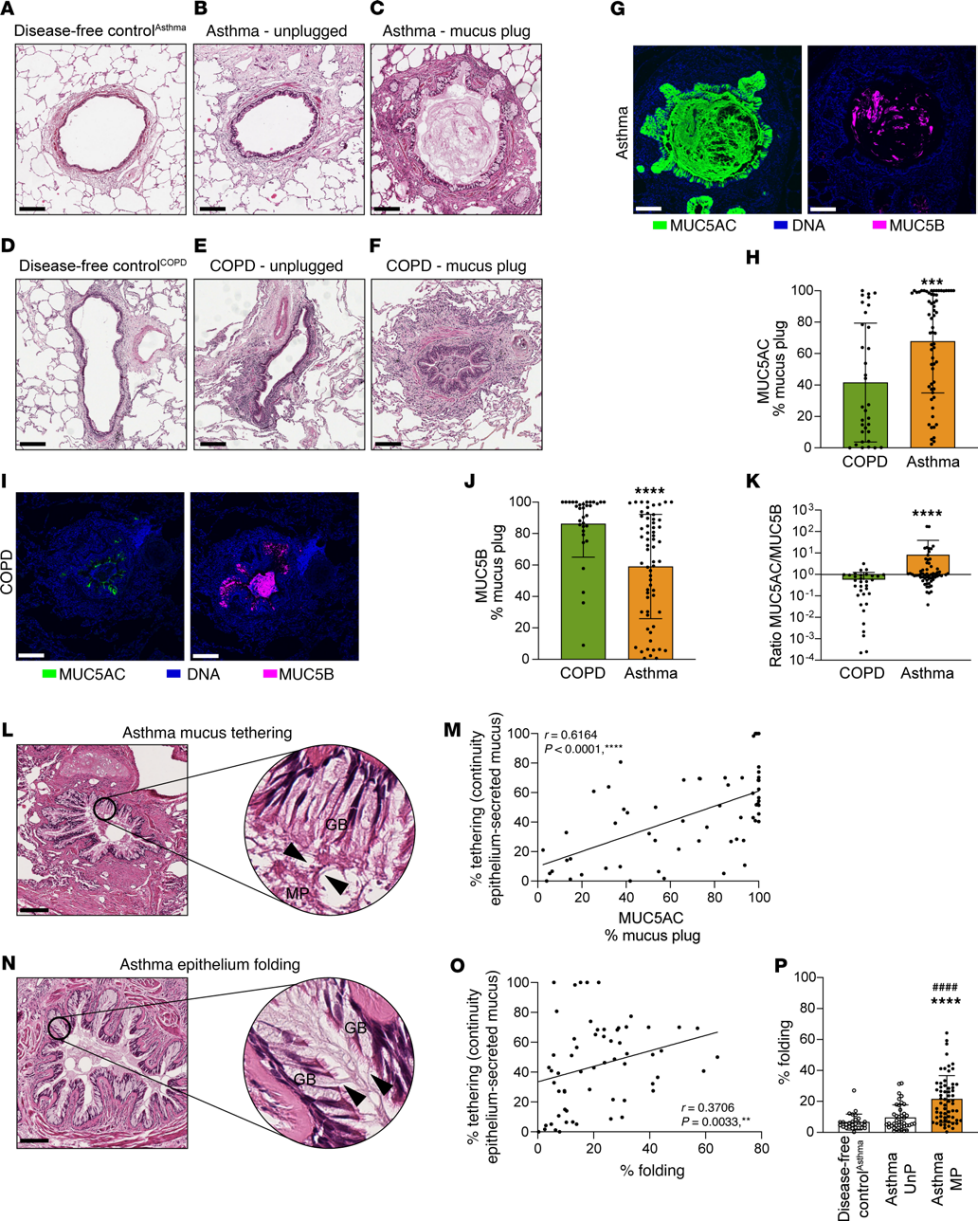
MUC5AC is the principal mucin in asthma mucus plugs, and epithelial mucus plug tethering correlates with extent of mucosal folding. (**A**) Lung disease–free control^Asthma^ airway (participant ID 7018). (**B**) Unplugged asthma airway (participant ID 7239). (**C**) H&E-stained asthma mucus plug (participant ID 7016). (**D**) Lung disease–free control^COPD^ airway (participant ID 7309). (**E**) Unplugged COPD airway (participant ID 7336). (**F**) H&E-stained COPD mucus plug (participant ID 7336). (**G**) Asthma mucus plugs stained for MUC5AC (green), MUC5B (magenta), and DNA (blue). (**H**) MUC5AC immunostaining in asthma mucus plugs is higher than in COPD mucus plugs. ***Significantly different from COPD, *P* < 0.001 (Mann-Whitney test). (**I**) COPD mucus stained for MUC5B (magenta), MUC5AC (green), and DNA (blue). (**J**) MUC5B immunostaining in asthma mucus plugs is lower than in COPD plugs. ****Significantly different from COPD, *P* < 0.0001 (Mann-Whitney test). (**K**) The MUC5AC/MUC5B ratio in asthma mucus plugs is higher than in COPD mucus plugs. ****Significantly different from COPD, *P* < 0.0001 (Mann-Whitney test). (**L**) Mucus strands (black arrowheads) connect the asthma mucus plug (MP) to the surface of goblet cells (GB) (participant ID 7187). (**M**) Mucus plug tethering percentage correlates with MUC5AC immunostaining in asthma mucus plugs (*n* = 61) (Spearman’s correlation). (**N**) Mucosal folds in mucus-plugged airways in asthma are rich in goblet cells (GB) and mucus is tethered to goblet cells at multiple points along the folds (black arrowheads) (participant ID 7237). (**O**) Mucus plug tethering percentage correlates with mucosal folding percentage in mucus plugs from patients with asthma (*n* = 61) (Spearman’s correlation). (**P**) Mucosal folding percentage is higher in mucus-plugged airways in asthma (asthma MP) than in unplugged airways in asthma (asthma UnP) or in lung disease–free control^Asthma^ airways. ****Significantly different from lung disease–free controls^Asthma^, *P* < 0.0001; ^####^significantly different from unplugged asthma airways, *P* < 0.0001 (Kruskal-Wallis test with Dunn’s correction). Scale bars: 200 μm.

We used histology and confocal imaging to characterize the mucin protein profile and histologic features of plugged and unplugged airways from patients with asthma (103 airways), patients with COPD (50 airways, disease control), and age-matched lung disease–free individuals acting as controls (lung disease–free control) airways (48 airways) (Figure 1). We found no mucus plugs in the airways of disease-free control lungs, whereas mucus plugs were identifiable in multiple airways from patients with asthma and COPD (Figure 2, A–F). We first analyzed the relative proportions of MUC5AC and MUC5B (the principal gel-forming mucins in the airways) in these mucus plugs. We found that mucus plugs from patients with asthma were rich in MUC5AC (Figure 2, G and H), whereas those from patients with COPD were rich in MUC5B (Figure 2, I and J), and the ratio of MUC5AC to MUC5B was significantly higher in asthma than in COPD (Figure 2K). We noticed that mucus plugs from patients with both asthma and COPD were tethered to the surface of goblet cells by mucin strands that connected the edge of the mucus plug to the epithelium (Figure 2L and Supplemental Figure 1, A and B; supplemental material available online with this article; https://doi.org/10.1172/JCI186889DS1). Using a metric of mucus tethering (percentage tethering, Supplemental Figure 2A) to quantify the degree of continuity between the mucus plugs and the epithelium, we found that percentage of tethering correlated positively and significantly with MUC5AC immunostaining in asthma but not in COPD (Figure 2M and Supplemental Figure 1C). We also noticed that the epithelium around the plugs was frequently buckled, forming bronchial mucosal folds that penetrated into the airway lumen to decrease the lumen area. The mucosal folds were rich in goblet cells, and we noticed that mucus secreted by these goblet cells was attached to the luminal mucus on both sides of the fold (Figure 2N and Supplemental Figure 1, D and E), which could have an effect to maintain the folds. Using a measure of bronchial mucosal folding (percentage folding, Supplemental Figure 2B), we quantified the difference between the size of the airway lumen and the basement membrane perimeter. We found that the percentage of folding correlated positively with percentage of mucus tethering in both asthma and COPD (Figure 2O and Supplemental Figure 1F). Specifically, the percentage of folding was higher in mucus-plugged airways from patients with asthma and COPD than in unplugged airways in these patient groups or in unplugged airways from lung disease–free individuals acting as controls (Figure 2P and Supplemental Figure 1G), indicating that the airway lumen is thereby smaller in mucus-plugged airways relative to their basement membrane perimeter.

### Spatial, single-cell proteomic characterization of mucus plugs and the airways in which they form.

**Figure 3:**
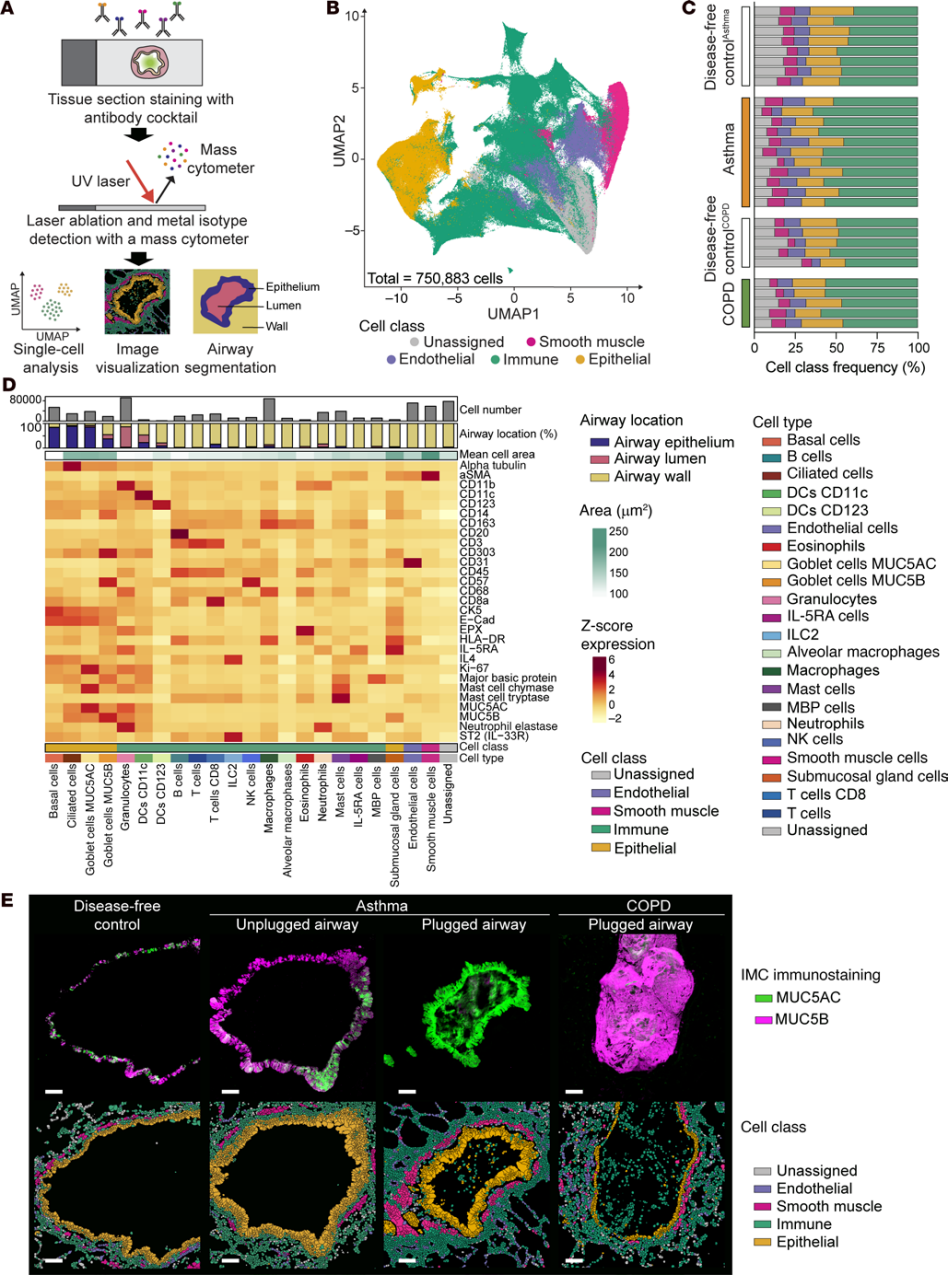
Spatial, single-cell characterization of mucus plugs and airways by imaging mass cytometry. (**A**) Schematic representation of the imaging mass cytometry (IMC) data generation and analysis pipeline. (**B**) Uniform manifold approximation and projection (UMAP) plot of all the cells identified in the IMC dataset, colored by cell classes. (**C**) Cell class frequency distribution among the different study participants: lung disease–free controls^Asthma^, patients with asthma, lung disease–free controls^COPD^, and patients with COPD. (**D**) Heatmap of marker expression for all cell classes and cell types. An additional heatmap shows cell area (in pixel [1 pixel = 1 μm^2^]). The 2 bar graphs show cell spatial categorization and the number of cells of each type, respectively. (**E**) Representative images of mucin immunostaining and cell class distribution after cell segmentation in a lung disease–free control airway (participant ID 7272), an asthma unplugged airway (participant ID 7239), an asthma mucus-plugged airway (participant ID 7188), and a COPD mucus-plugged airway (participant ID 6971). Scale bars: 100 μm.

In a subset of 117 airways from individuals with asthma, airways from individuals with COPD, and lung disease–free control airways, we used spatial single-cell imaging mass cytometry (IMC) to analyze the interaction of mucins with structural and immune cells in the mucus plugs and the airway wall (Figure 3A). In selecting this subset of airways, our objective was to enhance our understanding of the cellular environment surrounding asthma mucus plugs. Therefore, we prioritized the number of asthma airways (*n* = 55) over the number of COPD airways (*n* = 17), which we mostly used as a disease control group for mucus plug composition. In total, 750,883 cells comprising immune, epithelial, endothelial, and smooth muscle cells were assessed (Figure 3B). The frequency of the cell types varied among participants (Figure 3C), and the relatively high frequency of unassigned cells was because markers for common lung cells such as fibroblasts and alveolar epithelial cells were not included in the cell segmentation panel. A total of 23 different cell types were identified by marker expression (Figure 3D and Supplemental Table 3), and the most abundant cells were granulocytes (eosinophils and neutrophils), macrophages, smooth muscle cells, and endothelial cells (Figure 3D). We extracted the mean cell area measurement for each cell type, and we categorized cells according to their location in the airway lumen, airway epithelium, or airway wall (Figure 3D). Final images could be visualized through direct immunostaining or single-cell segmentation (Figure 3E and Supplemental Figure 3). In this way, we confirmed the confocal imaging data for expression of MUC5AC and MUC5B in mucus plugs and that the cell markers for airway epithelial cells, smooth muscle cells, and endothelial cells generated the expected airway images (Figure 3E). Spatial visualization of the 23 cell types provided details about their distribution across the different airways (Supplemental Figure 3).

### The airway epithelium of mucus-plugged airways in asthma is characterized by hyperplasia of basal cells and MUC5AC-positive goblet cells.

**Figure 4:**
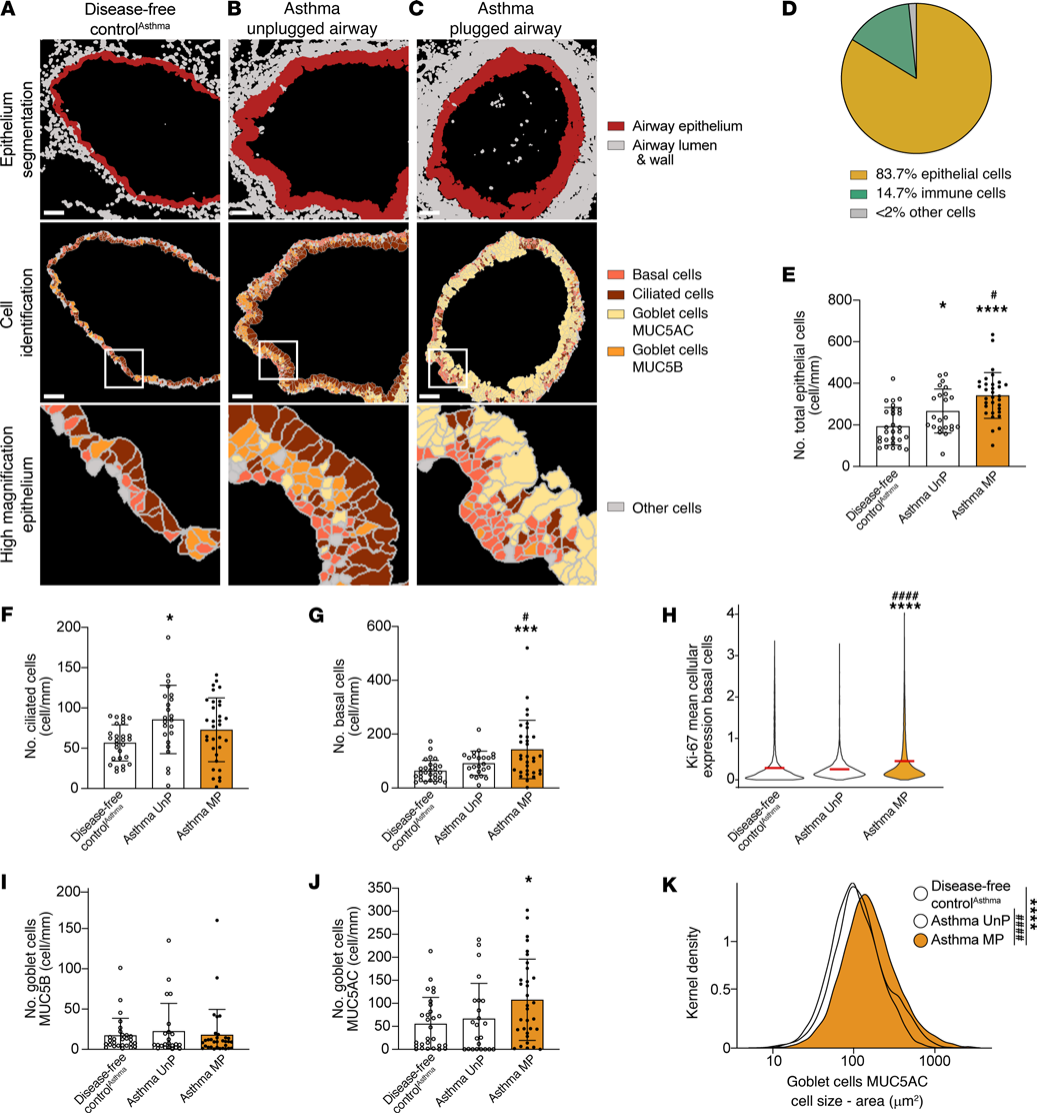
The airway epithelium of mucus-plugged airways in asthma is characterized by hyperplasia of basal cells and MUC5AC-positive goblet cells. (**A**–**C**) Representative cell-segmented images of segmented epithelium and epithelial cell types in a lung disease–free control^Asthma^ airway (participant ID 7234) (**A**), an asthma unplugged airway (participant ID 7239) (**B**), and an asthma plugged airway (participant ID 7016) (**C**). Scale bars: 100 μm. (**D**) Pie chart showing the cell class diversity of cells in asthma and disease-free control^Asthma^ airway epithelium. (**E**) Epithelial cell numbers are increased in asthma mucus-plugged airways. (**F**) Ciliated cell numbers are similar in lung disease–free control^Asthma^ and asthma plugged airways. (**G**) Basal cell numbers are increased in asthma mucus-plugged airways. (**H**) Basal cell proliferation is increased in asthma mucus-plugged airways (violin plot shows the Ki-67 mean cellular expression of basal cells. and the red lines indicate the average for each subgroup). (**I**) Epithelial goblet cells expressing MUC5B are similar in all patient groups. (**J**) Epithelial goblet cells expressing MUC5AC are increased in asthma airways occluded with mucus. (**K**) Kernel density plot showing a higher area for epithelial goblet cells expressing MUC5AC in mucus-plugged asthma airways than that in unplugged asthma airways and in lung disease–free control^Asthma^ airways. *Significantly different from lung disease–free control^Asthma^, *P* < 0.05; ***significantly different from lung disease–free control^Asthma^, *P* < 0.001; ****significantly different from lung disease–free control^Asthma^, *P* < 0.0001; ^#^significantly different from asthma unplugged airways, *P* < 0.05; ^####^significantly different from asthma unplugged airways, *P* < 0.0001 (ordinary 1-way ANOVA with Tukey’s correction).

To explore the pathology of the airway epithelium surrounding mucus plugs, single-cell IMC was used to assess the ciliated cells, basal cells, and goblet cells in individuals with asthma and lung disease–free individuals acting as controls (Figure 4, A–C). We found that the airway epithelium was composed of over 80% epithelial cells (Figure 4D), and the number of epithelial cells in asthma airways with mucus plugs was higher than in unplugged asthma airways or lung disease–free control airways (Figure 4E). While the number of ciliated cells was similar to that in lung disease–free individuals acting as controls (Figure 4F), the increase in total epithelial cell number in plugged airways was partially driven by an increase in the number of basal cells in these airways (Figure 4G). In addition, the basal cells in asthma airways with mucus plugs had higher proliferative activity, as evidenced by increased Ki-67 positivity (Figure 4H). Although the number of goblet cells expressing MUC5B was similar in asthma airways with mucus plugs, unplugged asthma airways, and lung disease–free control airways (Figure 4I), the number of goblet cells expressing MUC5AC was significantly higher in asthma airways occluded with mucus plugs (Figure 4J). The MUC5AC-expressing goblet cells were also larger in the mucus-plugged asthma airways (Figure 4K), indicating that MUC5AC-expressing goblet cells undergo both hyperplasia and hypertrophy. In COPD, the total number of epithelial cells in the airway epithelium of mucus-plugged airways was not significantly different than in unplugged COPD airways or in lung disease–free control airways (Supplemental Figure 4A).

### The airway wall of mucus-plugged airways in asthma is characterized by smooth muscle hyperplasia and ILC2 infiltration.

**Figure 5:**
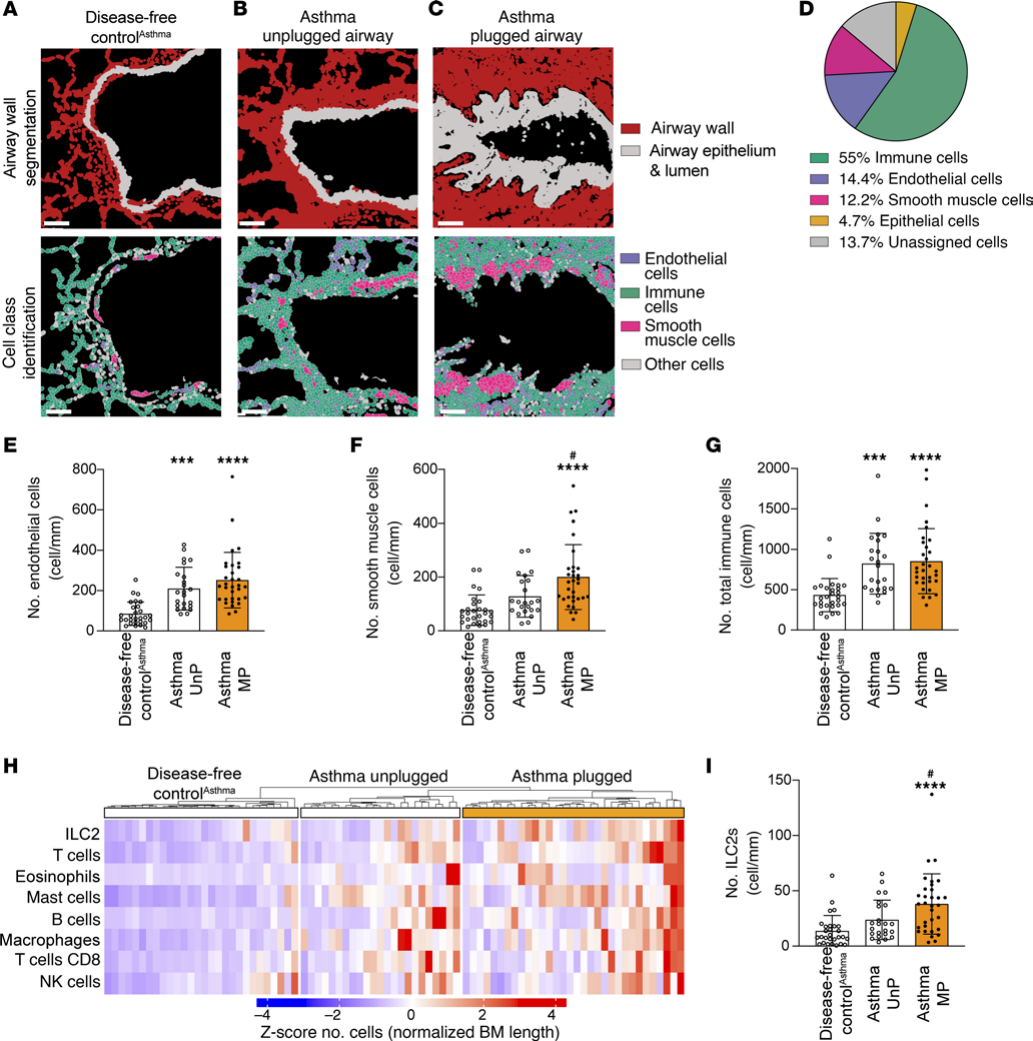
Smooth muscle cell hyperplasia and ILC2 infiltration characterize asthma airways occluded with mucus. (**A**–**C**) Representative cell-segmented images of airway wall categorization and cell class identification of a lung disease–free control^Asthma^ airway (participant ID 7018) (**A**), an asthma unplugged airway (participant ID 7238) (**B**), and an asthma plugged airway (participant ID 7237) (**C**). Scale bars: 100 μm. (**D**) Pie chart showing the cell class diversity of cells in asthma and disease-free control airway walls. (**E**) Endothelial cell numbers in asthma airway walls (unplugged and plugged) are higher than in lung disease–free control^Asthma^ airways. (**F**) Smooth muscle cell numbers in the walls of asthma airways plugged with mucus are higher than in unplugged asthma airway walls or in lung disease–free control^Asthma^ airway walls. (**G**) Immune cell numbers in airway walls in asthma (unplugged and plugged) are higher than in lung disease–free control^Asthma^ airways. (**H**) Heatmap of the *z* scores values of the immune cell types that are significantly higher in number in the airway wall of asthma unplugged and plugged airways compared with lung disease–free control^Asthma^ airways. (**I**) ILC2 numbers in the walls of asthma airways occluded with mucus are higher than in unplugged asthma airways and in lung disease–free control^Asthma^ airways. ***Significantly different from lung disease–free control^Asthma^, *P* < 0.001; ****significantly different from lung disease–free control^Asthma^, *P* < 0.0001; ^#^significantly different from asthma unplugged airways, *P* < 0.05 (ordinary 1-way ANOVA with Tukey’s correction).

To explore the pathology of the airway wall in which mucus plugs form in asthma, we applied IMC to characterize the structural cells and immune cells in the airway wall, excluding the airway epithelium (Figure 5, A–C). We found that most of the cells identified by IMC in the airway wall were immune cells, smooth muscle cells, and endothelial cells (Figure 5, A–D). Compared with lung disease–free control airways, endothelial cell numbers were increased in asthma airways with and without mucus plugs (Figure 5E), and smooth muscle cell numbers were specifically and significantly increased in asthma airways occluded with mucus plugs (Figure 5F). In addition, total immune cell number, comprising innate lymphoid type 2 cells (ILC2s), T cells, eosinophils, mast cells, B cells, macrophages, CD8^+^ T cells, and NK cells, was higher in asthma airways with and without mucus plugs than in lung disease–free control airways (Figure 5, G and H). Notably, ILC2 cells emerged from the IMC data as a cell type whose numbers were specifically increased in asthma airways occluded with mucus plugs (Figure 5I). In COPD, the number of endothelial cells, smooth muscle cells, or immune cells in the airway walls of mucus-plugged airways was not significantly different than in the airway walls of unplugged COPD airways or in lung disease–free control airways (Supplemental Figure 4, B–D).

### Mucus plugs are infiltrated with immune cells that are dual positive for markers of eosinophils and neutrophils.

**Figure 6:**
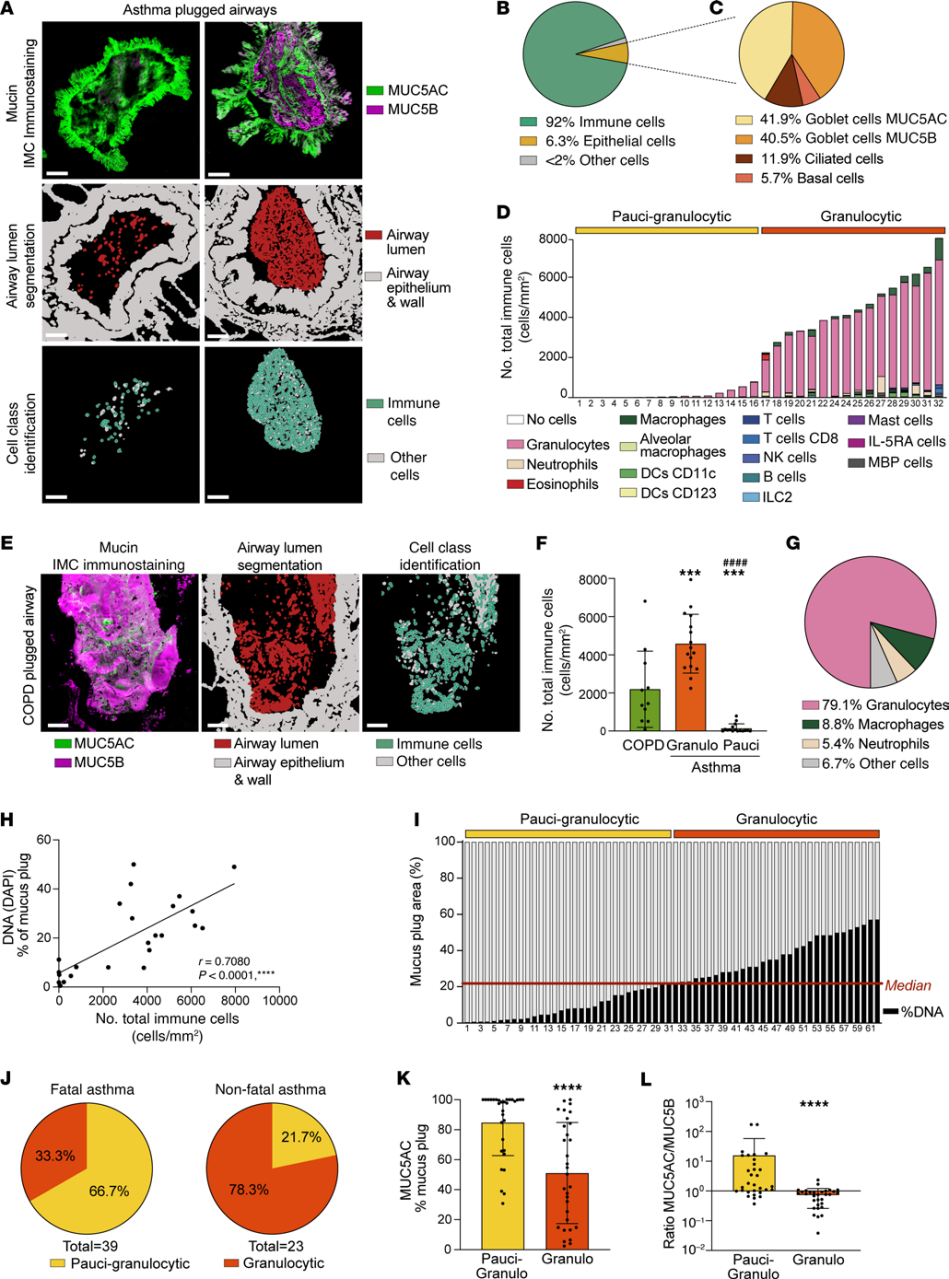
Paucigranulocytic mucus plugs rich in MUC5AC mucin are frequent in fatal asthma whereas granulocytic mucus plugs comprising a mix of MUC5AC and MUC5B mucins are frequent in nonfatal asthma. (**A**) Mucin immunostaining and cell-segmented images of lumen categorization and cell identification of 2 asthma plugged airways (participant IDs 7188 and 7298). Scale bars: 100 μm. (**B**) Pie chart showing the cell class diversity of cells infiltrating asthma mucus plugs. (**C**) Pie chart showing the epithelial cell type diversity of epithelial cells infiltrating asthma mucus plugs. (**D**) Diversity and prevalence of immune cell types infiltrating asthma mucus plugs, with granulocytes identified as the predominant cells. The median of total immune cell number was used to categorize mucus plugs as paucigranulocytic or granulocytic. (**E**) Mucin immunostaining and cell-segmented images of immune cells in a COPD plugged airway (participant ID 6967). Scale bars: 100 μm. (**F**) Immune cell infiltration of COPD mucus plugs is intermediate to that of asthma mucus plugs. ***Significantly different from COPD mucus plugs, *P* < 0.001; ^####^significantly different from asthma granulocytic plugs, *P* < 0.0001 (ordinary 1-way ANOVA with Tukey’s correction). (**G**) Pie chart showing the immune cell type diversity of cells infiltrating COPD mucus plugs. (**H**) Relationship between DNA immunostaining and the total immune cell number infiltrating airway lumen of mucus plugs analyzed by confocal imaging and IMC (*n* = 25) (Spearman’s correlation). (**I**) The median of DNA percentage was used to categorize mucus plugs as paucigranulocytic or granulocytic. (**J**) Paucigranulocytic mucus plugs are more frequent in fatal asthma, whereas granulocytic mucus plugs are more frequent in nonfatal asthma (*P* < 0.01, Fisher’s exact test). (**K**) MUC5AC immunostaining in paucigranulocytic mucus plugs is higher than in granulocytic mucus plugs. (**L**) The MUC5AC/MUC5B ratio is higher in paucigranulocytic plugs than in granulocytic plugs. ****Significantly different from paucigranulocytic plugs, *P* < 0.0001 (Mann-Whitney test).

To characterize the cellular profile of mucus plugs that occlude airways in asthma, we segmented mucus plugs to determine the number and type of immune cells that infiltrate them (Figure 6A). Among cells infiltrating asthma mucus plugs, immune cells predominated (92%), although there were some epithelial cells (6.3%) (Figure 6B), which were mainly goblet cells (Figure 6C). Among immune cells that infiltrate mucus plugs, cells double positive for eosinophil peroxidase (EPX) and neutrophil elastase (ELA2) were by far the most prevalent cells (Figure 6D). We used the “granulocyte” label for these EPX/ELA2 double-positive cells because of their dual expression of both eosinophil and neutrophil markers. We found that granulocytes were infrequent in some plugs and abundant in others, so we used the median split value for their number in plugs to categorize the plugs as either paucigranulocytic or granulocytic (Figure 6D). COPD mucus plugs, for their part, were also infiltrated by immune cells (Figure 6E) whose numbers were intermediate between those in asthma granulocytic and paucigranulocytic mucus plugs (Figure 6F). Similar to asthmatic plugs, the immune cells in COPD plugs were predominantly granulocytes (cells double positive for ELA2 and EPX, 79.1%), followed by macrophages (8.8%) and neutrophils (cells positive for ELA2 only, 5.4%) (Figure 6G and Supplemental Figure 4E).

### The frequency of paucigranulocytic and granulocytic mucus plugs differs in fatal and nonfatal cases of asthma.

To determine if the frequency of asthma mucus plug subtypes differs in fatal and nonfatal cases, we used the higher number of asthma mucus plugs that had been analyzed by histology and confocal imaging than by IMC (62 vs. 32), which provided more statistical power for the analysis. To categorize the plugs in the larger histology dataset as granulocytic or paucigranulocytic, we used percentage DNA staining as a measure of immune cell infiltration of the mucus plugs. There was a high correlation between percentage DNA staining and immune cells numbers in the mucus plugs analyzed by IMC (*r* = 0.7080 and *P* < 0.0001, Figure 6H), allowing the median percentage of DNA staining data to segregate 62 asthma mucus plugs into paucigranulocytic and granulocytic plugs (Figure 6I). In this way, we found that paucigranulocytic mucus plugs occur frequently in fatal asthma and that granulocytic mucus plugs are more frequent in nonfatal asthma (Figure 6J). Analyzing mucus plug subtypes on an individual participant basis, we found that fatal asthma cases mostly had higher numbers of paucigranulocytic plugs and nonfatal asthma cases mostly had higher numbers of granulocytic plugs (Supplemental Figure 5A). In addition, we noticed that paucigranulocytic plugs were characterized by high MUC5AC mucin content, whereas granulocytic plugs had a more balanced mix of MUC5AC and MUC5B mucins (Figure 6, K and L). Furthermore, paucigranulocytic plugs were characterized by higher amounts of tethering and folding than granulocytic plugs (Supplemental Figure 5, B and C). Taken together, these results suggest that paucigranulocytic mucus plugs result from acute degranulation of MUC5AC-positive goblet cells, whereas granulocytic plugs have a more complicated pathogenesis to account for their infiltration with granulocytes and their more balanced mix of MUC5AC and MUC5B mucins.

### Eosinophils interact with mucins secreted by IL-13–activated airway epithelial cells and undergo cytolysis.

**Figure 7:**
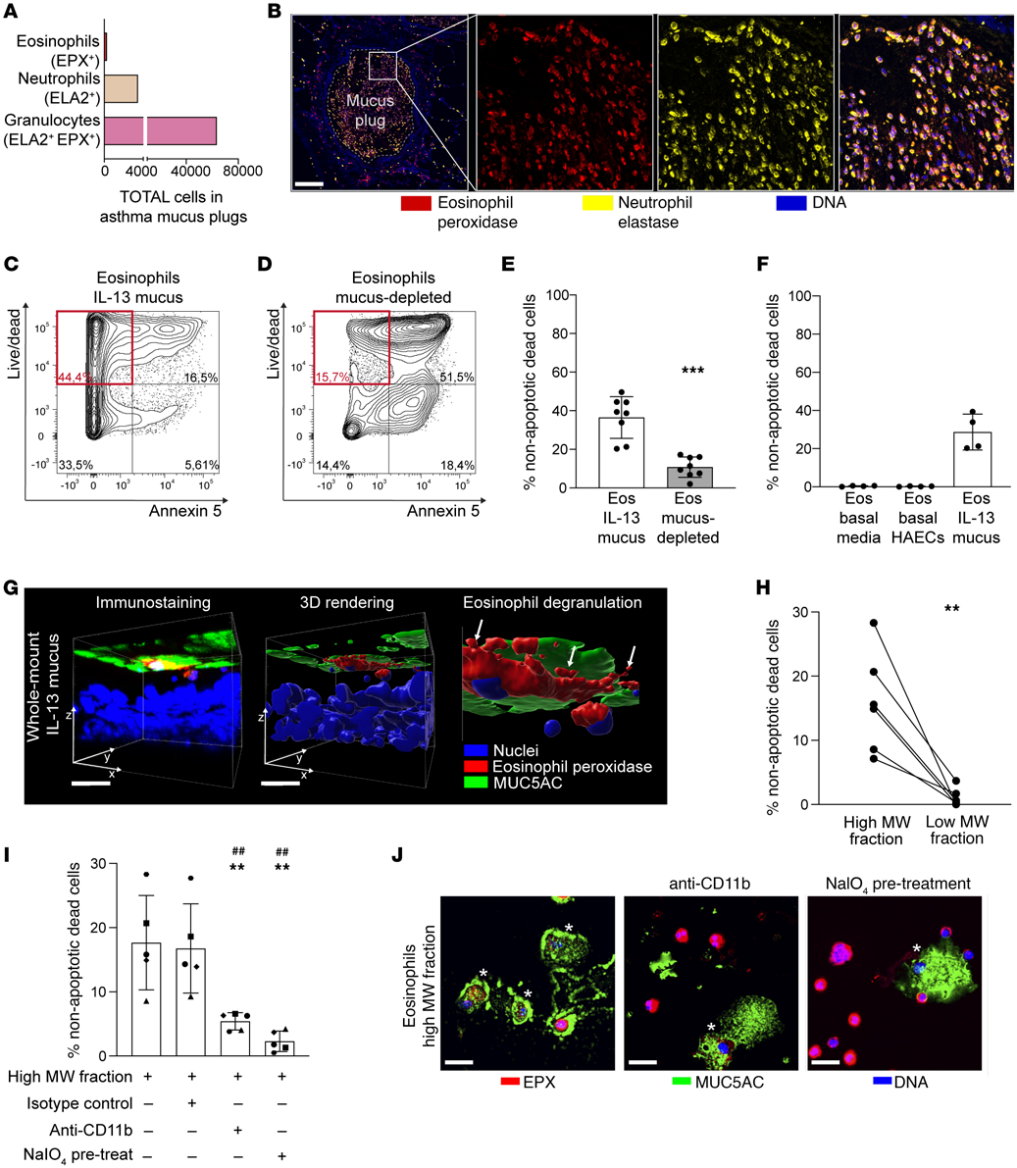
MUC5AC rich mucus secreted by IL-13–activated airway epithelial cells (HAECs) causes cytolytic degranulation of eosinophils. (**A**) Granulocytes dual positive for eosinophil peroxidase (EPX) and neutrophil elastase (ELA2) infiltrate asthma mucus plugs (IMC), although ELA2 single-positive neutrophils also occur. (**B**) Confocal imaging confirms cells in mucus plugs are double positive for EPX (red) and ELA2 (yellow) (participant ID 7233). DNA is shown in blue. Scale bar: 200 μm. (**C**) Nearly half (44.4%) of the eosinophils are dead^+^/annexin5^–^ (nonapoptotic dead cells) when overlaid on mucus layer of IL-13–activated human airway epithelial cells (HAECs). (**D**) Only 15.7% of eosinophils are dead^+^/annexin5^–^ cells when overlaid on mucus-depleted HAECs. (**E**) Nonapoptotic dead cell percentage is lower when eosinophils are overlaid on mucus-depleted HAECs (*n* = 8). ***Significantly different from eosinophils overlaid on IL-13 mucus, *P* < 0.001 (paired *t* test). (**F**) No eosinophils incubated in basolateral media of HAECs underwent nonapoptotic death (*n* = 4). (**G**) Representative immunostaining and 3D rendering of whole-mount cocultures of HAECs and eosinophils. EPX (red), MUC5AC (green), and nuclei (blue). White arrows mark eosinophil degranulation. Scale bars: 20 μm. (**H**) Nonapoptotic dead cell percentage is higher when eosinophils are incubated with high-molecular-weight versus low-molecular-weight-fraction (*n* = 6). **Significantly different from eosinophils incubated with high-molecular-weight mucus, *P* < 0.01 (paired *t* test). (**I**) Incubation of eosinophils with anti-CD11b or pretreatment of high-molecular-weight mucus with periodate (NaIO_4_) decreased nonapoptotic dead cell percentage. Symbols represent independent experiments (*n* = 5). **Significantly different from eosinophils incubated with high-molecular-weight mucus alone, *P* < 0.01 (ordinary 1-way ANOVA with Tukey’s correction). ^##^Significantly different from eosinophils incubated with high-molecular-weight mucus and isotype control, *P* < 0.01 (Tukey’s correction). (**J**) MUC5AC-coated degranulating eosinophils (asterisks) are visible when eosinophils are incubated with high-molecular-weight mucus. Incubation of eosinophils with anti-CD11b or pretreatment of high-molecular-weight mucus with periodate decreased the frequency of these MUC5AC-coated degranulating eosinophils. Scale bars: 20 μm.

The IMC analyses identified that immune cells infiltrating mucus plugs from patients with asthma predominantly coexpressed EPX and ELA2 (Figure 7A). A minority of immune cells exclusively expressed ELA2 and very few cells expressed only EPX (Figure 7A). To validate the IMC data, we used immunofluorescence methods to immunostain the mucus plugs for EPX and ELA2. This analysis confirmed that the majority of granulocytes infiltrating mucus plugs were double positive for EPX and ELA2 (Figure 7B). These data indicate that both eosinophils and neutrophils infiltrate the plugs, but the cells are somehow interacting in the mucin-rich environment of the plug. We hypothesized that eosinophils undergo degranulation with mucin exposure and that the extracellular EPX is bound and internalized by phagocytic neutrophils, as it has been previously reported (16). We therefore set out to determine whether mucins could cause eosinophil degranulation, by culturing human eosinophils in mucus secreted by human airway epithelial cells (HAECs) stimulated with IL-13 to recapitulate an allergic asthma environment (Supplemental Figure 6A). We found that exposure of eosinophils to epithelial cell mucus formed primarily of MUC5AC caused eosinophils to undergo nonapoptotic cell death, as evidenced by FACS data showing high numbers of cells in the dead cell fraction and low expression of annexin-5 (Figure 7C). Notably, the percentage of eosinophils undergoing nonapoptotic cell death was significantly lower when eosinophils were overlaid on mucus-depleted epithelial cells (Figure 7, D and E). Although IL-13 stimulation is also known to change the basolateral secretions of epithelial cells (17), we found that basolateral secretions of IL-13–stimulated cells did not change eosinophil viability (Figure 7F and Supplemental Figure 6, B and C), indicating that the induction of nonapoptotic cell death is specific to apical secretions. Nonapoptotic death of eosinophils associated with degranulation in epithelial mucus could suggest that cytolysis has occurred. Eosinophil degranulation during cytolytic cell death is distinct from classical exocytosis where no cell death occurs or apoptosis where programmed cell death occurs without degranulation (18). To confirm that eosinophils degranulate and release EPX in MUC5AC-rich mucus, we used 3D renderings of whole-mount and apical washes of the epithelial and eosinophil cocultures. In this way, we showed that eosinophils degranulate and release EPX in the MUC5AC-rich mucus layer (Figure 7G, Supplemental Figure 6D, and Supplemental Video 1). In addition, we noted that eosinophils exposed to apical mucus are coated in MUC5AC (Supplemental Figure 6C), suggesting a direct, possibly contact-dependent, interaction between eosinophils and mucins.

### Mucins induce eosinophil cytolytic degranulation in a mechanism mediated by mucin glycans and eosinophil CD11b.

To investigate the mechanism of interaction between mucus and eosinophils, we used ultrafiltration to separate the high- and low-molecular-weight fractions of apical secretions from IL-13–treated HAECs (Supplemental Figure 6E). We found that only the high-molecular-weight (mucin-rich) fraction induced eosinophil nonapoptotic cell death (Figure 7H and Supplemental Figure 6, F and G). In considering how mucins might cause eosinophil cytolysis, we noted prior research that shows that fibrinogen is a specific trigger for cytolytic eosinophil degranulation and that the mechanisms is integrin (CD11b) mediated (19). Other studies have also implicated CD11b in mechanisms of eosinophil adhesion to polymers and pathogens (20, 21). In addition, any mechanism of mucin-related cell activation needs to consider the role of mucin glycans, since mucins are heavily glycosylated and these glycans have important signaling functions (22). We therefore tested if inhibiting CD11b or removing the glycan coat of mucins would reduce the cytolytic effect of mucus on eosinophils. We found that inhibition of CD11b on eosinophils significantly decreased eosinophil nonapoptotic death when the eosinophils were exposed to the high-molecular-weight fraction of epithelial cell mucus (Figure 7I and Supplemental Figure 6, H and I). In addition, we found that eosinophils exposed to high-molecular-weight fraction of epithelial cell mucus that had been treated with periodate to remove glycans also showed significantly decreased eosinophil nonapoptotic death (Figure 7I and Supplemental Figure 6, H and K). Furthermore, using microscopy, we found that eosinophils incubated with high-molecular-weight mucus are heavily coated with MUC5AC and show a degranulating phenotype (Figure 7J). CD11b inhibition on eosinophils or periodate treatment of the high-molecular-weight fraction of the mucus decreased the number of these degranulating MUC5AC-coated eosinophils by 55% and 80%, respectively (*P* < 0.0001 for both reductions vs. control). Taken together, these findings demonstrate that eosinophils undergo cytolytic degranulation when in contact with MUC5AC-rich airway mucus in a glycan- and CD11b-dependent manner, making the released EPX available to be bound and internalized by neutrophils.

### Granulocytic mucus plugs are infiltrated by extracellular traps.

**Figure 8:**
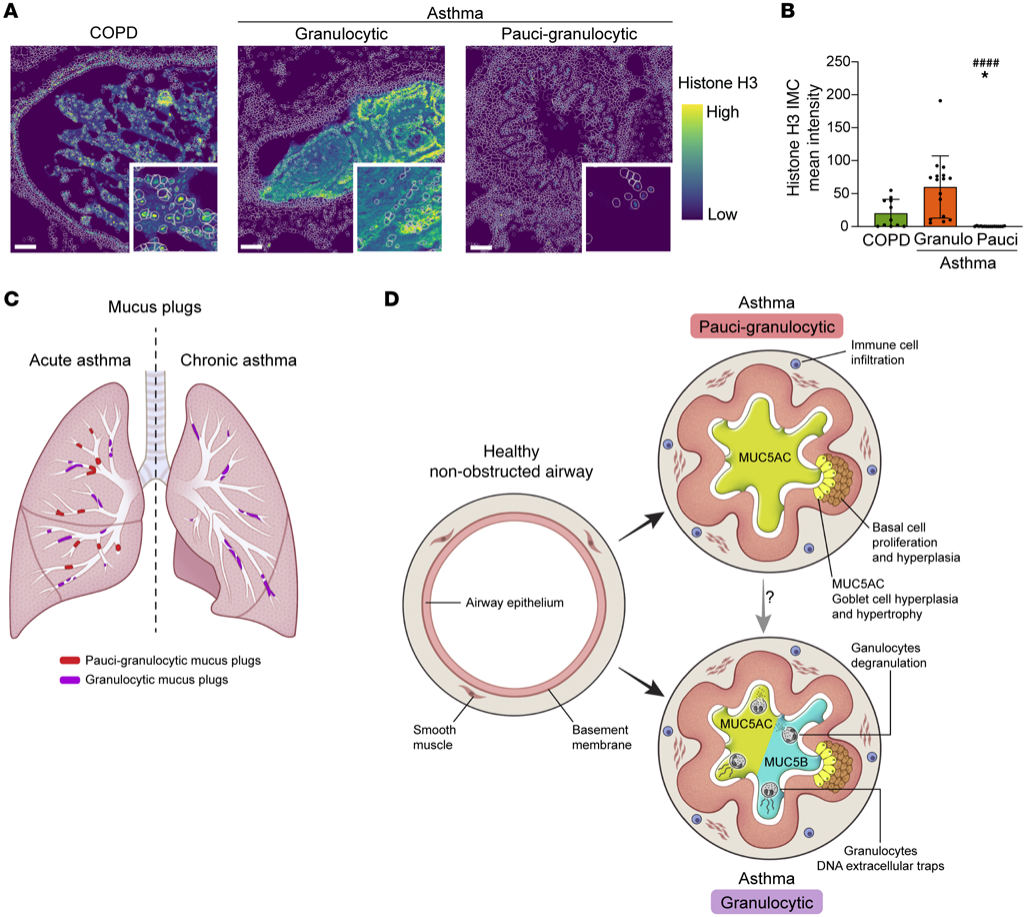
Granulocytic mucus plugs have increased numbers of extracellular DNA traps. (**A**) COPD mucus plugs (participant ID 6968) and granulocytic asthma mucus plugs (participant ID 7239) show extracellular histone H3 immunostaining. Paucigranulocytic mucus plug (participant ID 7188) shows histone H3 immunostaining only within the cell boundaries. Scale bar: 100 μm. (**B**) Mean intensity of histone H3 immunostaining in COPD mucus plugs and asthma granulocytic mucus plugs is higher than in asthma paucigranulocytic mucus plugs. *Significantly different from COPD mucus plugs, *P* < 0.05 (Kruskal-Wallis test with Dunn’s correction). ^####^Significantly different from asthma granulocytic mucus plugs, *P* < 0.0001 (Kruskal-Wallis test with Dunn’s correction). (**C**) Paucigranulocytic mucus plugs are more common in acute asthma than in chronic asthma. (**D**) Schematic summary of findings for mucus plugs from patients with asthma. Asthma mucus plugs form in airways that are inflamed and remodeled, displaying features such as folding of the epithelium and hyperplasia of smooth muscle cells, basal cells, and goblet cells. Two subtypes of mucus plugs are identified in asthma: paucigranulocytic, which are high in MUC5AC and low in cellular infiltration, and granulocytic, which have a balanced mix of MUC5AC and MUC5B and high numbers of infiltrating granulocytes. Granulocytes in these plugs tend to degranulate and form DNA extracellular traps. Paucigranulocytic mucus plugs likely result from acute goblet cell degranulation while granulocytic mucus plugs may be a subset of acute mucus plugs that fail to resolve and become chronic.

The high DNA levels and prominent infiltration of mucus plugs by granulocytes prompted us to explore if granulocytic mucus plugs are infiltrated with extracellular DNA traps. Using the IMC dataset, we found that mucus plugs from patients with COPD and granulocytic mucus plugs from patients with asthma showed extracellular histone H3 immunostaining, while the paucigranulocytic mucus plugs exhibited histone H3 staining exclusively within the cell boundaries (Figure 8A). The median intensity of histone H3 immunostaining in the airway lumen was significantly higher in COPD mucus plugs and asthma granulocytic mucus plugs than in asthma paucigranulocytic mucus plugs (Figure 8B). Asthma granulocytic mucus plugs are therefore characterized by high number of granulocytes that release extracellular traps and by a mucin profile that includes both MUC5AC and MUC5B mucins. In contrast, asthma paucigranulocytic plugs are characterized by low numbers of granulocytes and by a mucin profile dominated by MUC5AC.

## Discussion

To develop targeted therapies that effectively address mucus plugs, it is essential to explore and understand the underlying biological environment that contributes to rapid mucus plug formation in acute severe asthma and to the mechanisms that sustain persistence of mucus plugs in chronic asthma. Here, we applied immunohistochemistry and IMC to characterize the mucins and immune cells in airway mucus plugs in lung tissue from patients with asthma (fatal and nonfatal cases), providing information about mucus plug formation and persistence. More than 750,000 cells across multiple airways were resolved, enabling spatial analysis of structural cells, immune cells, and epithelial cells in the airway wall as well as infiltrating immune cells in mucus plugs. Spatial, single-cell analyses showed that mucus-plugged airways were remodeled and characterized by smooth muscle hyperplasia, immune cell inflammation, and hyperplasia of basal cells and MUC5AC-positive goblet cells. Interestingly, immune cell infiltration of mucus plugs consisted primarily of cells that were dual positive for markers of eosinophils and neutrophils, and these granulocytes were infrequent in some plugs and abundant in others. Paucigranulocytic plugs were more common in fatal asthma, and granulocytic plugs were more common in nonfatal asthma. The paucigranulocytic plugs were MUC5AC rich, whereas granulocytic plugs had a more balanced mix of MUC5AC and MUC5B and showed extracellular DNA traps. Taken together, our results imply that the mucus plugs that form during acute asthma exacerbations occur because of acute goblet cell degranulation in remodeled airways and that mucus plugs that are found in chronic asthma may represent nonresolved acute plugs that become infiltrated with granulocytes to create a self-sustaining niche in the airway lumen.

The mucus plugs from patients with asthma occurred in airways that were characterized by mucosal folds that decreased the airway lumen size. The mechanism of the mucosal folding or buckling is thought to be airway smooth muscle contraction (23, 24), but it is possible that mucosal inflammation also has a direct or indirect role. In addition, we provide here data that suggest that mucus plug tethering to the epithelial surface could be a mechanism of causation or persistence of mucosal folds. Specifically, as a causal mechanism, it is possible that mucin tethers exert forces on the airway mucosa to fold it. As a mechanism of persistence, we show how abundant goblet cells in the folds secrete mucins that tether to the luminal mucus. This tethering occurs from both sides of the folds and could have an effect to maintain the epithelium in a folded state. Mucus plug–related epithelial folding thus represents a mechanism, other than direct occlusion, by which mucus plugs could decrease airway lumen size and airflow in asthma. Another recently highlighted consequence of epithelial buckling and epithelial mucosal folding in asthma is extrusion of epithelial cells (25). We found evidence of extrusion of goblet cells, because goblet cells were a component of the mucus plugs, although the frequency of goblet cells in the plugs is small relative to the number of granulocytes.

In characterizing the cellular features of the airway wall and epithelium surrounding mucus plugs in asthma, we found that these plugs form in airways that are remodeled in both subepithelial and epithelial layers. First, hyperplasia of smooth muscle cells was evident, which could mediate narrowing of the airway lumen to enable occlusion with mucus plugs. Second, there was hyperplasia and hypertrophy of MUC5AC-positive goblet cells, which could prime the airways for acute and chronic mucin degranulation in response to environmental cues. Our findings of higher numbers of MUC5AC-positive goblet cells in mucus-plugged airways confirmed and extended prior findings of MUC5AC overexpression in the airways in fatal and nonfatal asthma (10, 26). Third, there is basal cell hyperplasia and proliferation. Increases in basal cells have previously been reported in asthma (27, 28), but we report here that basal cell number and proliferation is highest in asthma airways that are plugged with mucus. Basal cells differentiate into goblet cells when SAM-pointed domain-containing Ets-like factor (SPDEF) is upregulated (29), and such upregulation occurs with IL-13 activation (30, 31). The increased number of ILC2 cells in mucus-plugged airways provides a local source of IL-13 and other type 2 cytokines. Our data thus reveal a specific airway environment around mucus plugs that primes these airways for mucus plug formation.

In characterizing the cellular features of the mucus plug themselves in asthma, we found that the predominant infiltrating cells were granulocytes that were frequently dual positive for eosinophil peroxidase and neutrophil elastase. In exploring the reasons for this dual positivity, we uncovered that airway mucus can cause eosinophil cytolysis and the release of EPX. Specifically, our data reveal that eosinophils interact with MUC5AC to induce cytolytic degranulation in a contact-dependent mechanism that involves mucin glycans and CD11b expression on eosinophils. Notably, eosinophil cytolysis and degranulation occur with exposure to soluble fibrinogen and the mechanism is CD11b dependent (19). Large glycoproteins such as mucins and fibrinogen may therefore share a common CD11b pathway to induce cytolytic degranulation of eosinophils with release of EPX. Because neutrophils can uptake EPX (16), we interpret our data overall to mean that the dual-positive granulocytes in the mucus plugs are neutrophils that have scavenged EPX from eosinophils that have undergone cytolysis in the mucin-rich environment of the plug.

The number of granulocytes infiltrating the mucus plugs varied from few to many, and paucigranulocytic plugs were more common in fatal asthma whereas granulocytic plugs were more common in nonfatal asthma. The relatively acellular and MUC5AC-rich profile of mucus plugs in fatal asthma is consistent with their formation because of acute degranulation of MUC5AC-positive goblet cells. Such acute degranulation could occur in response to environmental exposure known to cause acute severe asthma attacks, including exposure to respiratory viruses, aeroallergens, wood smoke, or a range of chemicals (32). We have previously reported that ELA2 has a role in turning over mucus plugs in acute severe asthma (33). The relative lack of granulocytes in many of the fatal asthma mucus plugs reported here indicates that these plugs can form without granulocyte assistance, perhaps as a result of very high MUC5AC protein levels that can self-form mucus plugs. And because the elastase turnover effect takes hours to days, it is possible that the patients with fatal asthma described here died before neutrophils could infiltrate the plugs and elastase could lyse them. We have also previously reported in chronic severe asthma that EPX drives oxidant acid–induced mucin cross-linking and mucus plug formation (5). The abundance of granulocytes in many of the nonfatal asthma mucus plugs may mean that these plugs persist with granulocyte assistance that involves peroxidase-mediated cross-talk with mucins.

The granulocytic plugs in nonfatal asthma were characterized by a balanced mixture of MUC5AC and MUC5B mucins and the presence of extracellular DNA traps. The mucus plugs in nonfatal COPD cases had a similar profile (granulocytic, enrichment in MUC5B content, and presence of extracellular DNA traps), indicating that this mucus plug phenotype is a feature of airway mucus plugs in chronic asthma and chronic COPD. The persistence of mucus plugs for long periods of time in chronic forms of asthma and COPD is a relatively recent discovery (5, 6, 34, 35), and the mechanisms underlying this persistence is unknown. Radiographic studies of patients with acute but nonfatal exacerbations of asthma indicate that mucus plugs usually resolve during recovery (36), but it is possible that some plugs do not resolve and persist for long periods, much as incomplete thrombus resolution following acute thrombus formation is thought to account for persistent clots in chronic thromboembolic pulmonary disease (37). The fact that patients with asthma with persistent mucus plugs on CT are characterized by an exacerbation-prone phenotype (6) supports the possibility that chronic mucus plugs may derive from acute mucus plug formation in these patients.

The infiltration of mucus plugs with granulocytes creates a specific cross-talk environment in which mucus plugs persist. As described above, peroxidases from these cells may interact directly with the mucus hydrogel to increase the density of mucin disulfide cross-links (5, 38). In addition, signaling molecules from granulocytes may interact with the airway epithelium to upregulate mucin gene expression (39), and extracellular DNA traps from granulocytes can serve to facilitate the interaction of these cells with epithelial cells and the mucus gel, much like their role in linking inflammation and thrombosis (40). Furthermore, Charcot-Leyden crystals (consisting of galectin-10 protein) released by eosinophils can stimulate neutrophilic airway inflammation and drive goblet cell metaplasia (41). Finally, occlusive mucus plugs can cause local airway hypoxia that induces metabolic and transcriptional changes in epithelial cells to upregulate mucin genes (MUC5B in particular) (15). Hypoxia could also increase granulocyte survival and amplify granulocyte-driven airway remodeling (42–44). Also relevant here is that, although mucins have multiple antiinflammatory properties, these proteins can also regulate ATP release from epithelial cells to promote inflammation (12). Thus, there are multiple possible cross-talk pathways to explain a self-sustaining mucin-granulocyte-epithelial cell niche that promotes persistence of mucus plugs.

In integrating the data from the analysis of mucus plugs from patients with asthma, we conclude that mucus plugs in fatal asthma represent a combination of newly formed mucus plugs (acute plugs) and chronic plugs that together cause widespread occlusion of the airways to result in acute respiratory failure (Figure 8C). The acute plugs are relatively acellular and MUC5AC-rich and most likely to form as a consequence of acute degranulation of goblet cells in remodeled and inflamed airways. The chronic mucus plugs are more frequently infiltrated with granulocytes, and they may arise from nonresolution of acute mucus plugs (Figure 8D). These data and concepts provide a framework for tackling mucus plug pathology that supports treatments that target airway remodeling and mucin gene dysregulation as well as those that target the biological pathways that drive granulocyte trafficking to mucus plugs in the airway lumen and the formation of extracellular DNA traps.

## Methods

### Sex as a biological variable.

Lung tissue samples from male and female individuals were included in the study.

### Human biospecimens and demographic data.

Formalin-fixed, paraffin embedded lung tissue sections from nontransplantable donor lungs of patients with asthma (*n* = 14), patients with very-severe COPD (*n* = 10), and healthy individuals acting as controls (*n* = 14) were obtained from the James Hogg Lung Biobank (45). Of the patients with asthma, 9 had died of an asthma attack and 5 had died of a nonasthma-related cause but had asthma during their lifetime. Because the patients with asthma were younger than the patients with COPD, we analyzed lung tissue from 2 control groups who had died from nonpulmonary causes. One group (comprising participants without lung disease who were compared with patients with asthma [lung disease–free control^Asthma^]) had lung tissue from younger patients (*n* = 8), and the other group (comprising participants without lung disease who were compared with patients with COPD [lung disease–free control^COPD^]) had lung tissue from older patients (*n* = 6) (Table 1). Ethnicity data are missing for 1 participant due to incomplete records.

### Airway section analyses.

Lung tissue sections of 5 μm stained with H&E were examined under a light microscope to identify airways mucus plugs. Where possible, at least 1 plugged airway and 1 unplugged airway from the same donor were analyzed. Large airways (diameter >2 mm) and small airways (diameter <2 mm) were included in the study (Supplemental Table 1). Different 5 μm lung tissue sections were analyzed by H&E staining, immunofluorescence, and IMC, as detailed in the Supplemental Methods, and summarized below.

### Quantification of mucins and DNA.

Tissue immunostaining was quantified using ImageJ software (NIH), with maximum intensity projection images used to measure the lumen area occupied by mucus plugs and calculate the percentages positive for DNA, MUC5AC, or MUC5B (Supplemental Figure 7, A–C). Additional details are available in the Supplemental Methods.

### Quantification of tethering of mucus plugs to the airway epithelium and of the amount of folding of the airway epithelium.

Tethering and folding outcomes were measured on digitally scanned H&E-stained lung tissue sections (×20, Aperio Digital Pathology Scanner, Leica), and files were analyzed with the QuPath software (46). To measure mucus tethering to the epithelium, a line was drawn around the lumen perimeter, and then a line was placed in each region of the mucus plug that was presenting continuity between the secreted mucus and the epithelium to allow calculation of the percentage of the lumen that had mucus plugs tethered to it (Supplemental Figure 2A). To measure epithelium folding, a line was drawn around the lumen perimeter, and then a line was placed on the basement membrane perimeter to allow calculation of folding as the difference between the two perimeters divided by the basement membrane perimeter (Supplemental Figure 2B).

### IMC.

A total of 165 regions of interest (ROIs) were scanned with Hyperion plus (Standard BioTools). These regions represented a total of 117 airways that were selected among patients with asthma (*n* = 11, mucus plugs = 32, unplugged airways = 23), lung disease–free control^Asthma^ participants (*n* = 8, airways = 28), patients with COPD (*n* = 5, mucus plugs = 11, unplugged airways = 6), and lung disease–free control^COPD^ participants (*n* = 5, airways = 17). The number of ROIs is higher than the number of airways analyzed because some airways were larger than the Hyperion capture area and required multiple ROIs to cover their size. To characterize the cellular environment of each airway, a custom panel of 35 antibodies was used (Supplemental Table 2) as detailed further in the Supplemental Methods.

### IMC data analysis.

The IMC pipeline for data analysis and cell segmentation was adapted from Windhager et al. (47) and is summarized in Supplemental Figure 8 and detailed in the Supplemental Methods. Briefly, mcd files generated by the Hyperion plus apparatus were preprocessed with the readimc Python package, and single-cell segmentation was done following the IMC segmentation pipeline. Ilastik software was used for pixel training to classify each pixel as cytoplasm, nuclei, or background. The CellProfiler software was used to generate cell masks and to generate single-cell information such as mean intensity per channel and spatial neighbor information. All the downstream analyses were done in R, using the imcRtools package for single-cell analysis and the cytomapper package for image visualization. Four cell markers (CD4, BCL-XL, IL-3RB, and BCL-2) provided poor staining quality and were excluded from analysis (Supplemental Figure 9A). Batch effect was corrected using the Seurat package (Supplemental Figure 9, B and C). Finally, the Phenograph algorithm was used for unsupervised clustering, and 29 markers were used for cell type identification (Supplemental Table 3).

### HAECs and eosinophils cocultures.

Details regarding the methods for coculturing HAECs and human blood eosinophils are provided in the Supplemental Methods.

### Statistics.

IMC data were analyzed on R version 4.4.0, using imcRtools for single-cell analysis and cytomapper for image visualization. Statistical analyses were performed using Prism 9. A 2-tailed Student’s *t* test was used for 2-group comparisons of parametric data, and Mann-Whitney *U* test was used for 2-group comparisons of nonparametric data. In the case of parametric multiple group comparisons, 1-way ANOVA test was used, followed by Tukey’s correction. In the case of nonparametric multiple group comparisons, Kruskal-Wallis test was used followed by Dunn’s correction. All bar plots are shown as mean ± SD. Correlation between nonparametric variables was assessed using Spearman’s correlation. The statistical tests used are specified in the figure legend. *P* values lower than 0.05 were considered as significant.

### Study approval.

For the lung tissue samples analyzed in this study, the patients or their next of kin provided informed consent, and the study was approved by the Research Ethics Board of the University of British Columbia (H13-02173).

### Data availability.

The values corresponding to each data point in the different graphs can be found in the Supporting Data Values file. The combined single-channel TIFF files and analysis codes are available at https://doi.org/10.6084/m9.figshare.26972767.v1

## Author contribution

MAL and JVF conceived the study. MAL conducted the experiments and data analysis, including bioinformatics. AH, MAF, ARC, and CXY contributed to data collection and data analysis. TLH is the director of the James Hogg Lung Biobank and contributed to data collection as well as study design. MAL and JVF wrote the first draft of the manuscript, and all authors reviewed and approved the final version of the manuscript.

## Supplementary Material

Supplemental data

ICMJE disclosure forms

Supplemental video 1

Supporting data values

## Figures and Tables

**Table 1:**
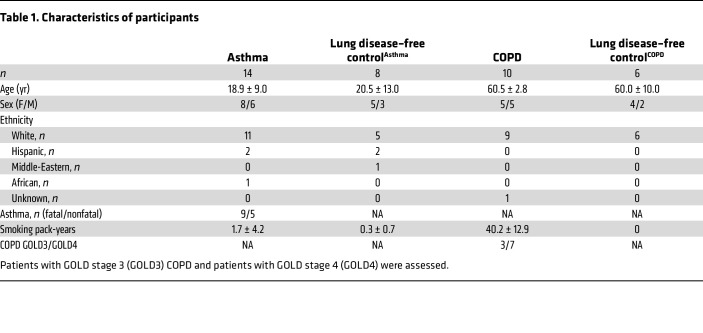
Characteristics of participants

## Version 1 03/17/2025

Electronic publication
