# Supplementary material for: Cellular and molecular features of asthma mucus plugs provide clues about their formation and persistence
Source: J Clin Invest. 2025 Mar 17;135(6):e186889. doi: 10.1172/JCI186889 (PMC11910225; doi:10.1172/JCI186889)
Supplement: Supplemental data [file jci-135-186889-s170.pdf]

Cellular and molecular features of asthma mucus plugs provide clues about their formation and persistence

Authors

Maude A. Liegeois[1], Aileen Hsieh[2,3] May Fouadi[2,3], Annabelle R. Charbit[1], Chen Xi Yang[2,3], Tillie-Louise Hackett[2,3] & John V. Fahy[1,4*]

Affiliations

[1]Cardiovascular Research Institute, University of California, San Francisco, San Francisco, CA, USA.

[2]Centre for Heart Lung Innovation, Vancouver, British Columbia, Canada.

[3]Department of Anesthesiology, Pharmacology and Therapeutics, University of British Columbia, British Columbia, Canada

[4]Division of Pulmonary, Critical Care, Allergy and Sleep Medicine, University of California, San Francisco, San Francisco, CA, USA.

*** Corresponding authors**

John V. Fahy, M.D., M. Sc.,

Division of Pulmonary and Critical Care Medicine, Department of medicine,

University of California San Francisco,

Room 1303, Health Science East, 513 Parnassus Avenue

San Francisco, CA 94143

E-mail : John.Fahy@ucsf.edu

Material and methods

Immunofluorescence microscopy

The tissue sections were deparaffinized in xylene, followed by slide-rehydration through a graduated ethanol series and water. Antigen retrieval was performed with a citrate-based antigen unmasking solution (Vector laboratories). Cells were permeabilized with triton X-100 0.1% buffer (Sigma-Aldrich) followed by an incubation in a phosphate-buffered saline (PBS) solution with 10% goat serum (Gibco) and 0.05 % Tween 20 (Sigma-Aldrich) to prevent unspecific antibody binding. The tissue sections were stained overnight at 4°C with a mouse monoclonal anti-MUC5AC antibody (1:300, 45M1, ThermoFisher) or a mouse monoclonal anti-MUC5B antibody (1:200, 6F10-E4, Abcam). The next day, a secondary antibody, Cy-3 goat anti-mouse (1:300, Jackson Immunoresearch), was added for 2 hours. 4',6-diamidino-2-phenylindole (DAPI) (1:1000, Calbiochem) was used to stain the nuclei. After staining, a series of 20 optical sections (Z-stacks) were acquired with an Olympus Fluoview 10i laser scanning confocal microscope at 10x magnification using the same excitation laser values for all slides having the same immunostaining.

In the case of eosinophil peroxidase (EPX) and neutrophil elastase (ELA2) co-staining, IHC Antigen Retrieval Solution (Invitrogen) was used for antigen retrieval. The tissue sections were stained overnight at 4°C with a mouse monoclonal anti-ELA2 antibody (1:100, 950317, R&D) and a rabbit polyclonal anti-EPX antibody (1:200, Diagnostics Development). The next day, a Cy-3 goat anti-mouse and a Cy-5 goat anti-rabbit (1:300, Jackson Immunoresearch) were used as secondary antibodies. Images were acquired

with the Olympus Fluoview 10i laser scanning confocal microscope at 10x and 60x (oil immersion objective) magnification.

Quantification of mucins and DNA

Tissue staining was quantified using the ImageJ software. First, a maximum intensity projection image was generated for each Z-stack and immunostaining, and the brightfield images were used to measure the lumen area occupied by mucus plugs (Supplemental Figure 7A). The threshold intensity was set using the triangle algorithm (1), which produced binary images, and only black area within the lumen and excluding the airway wall were considered as mucus plug area (Supplemental Figure 7A). The lumen area positive for DNA or mucins was divided by the total mucus plug area to calculate the percentage of the mucus plug positive for DNA, MUC5AC, or MUC5B (Supplemental Figure 7B). The ratio of MUC5AC to MUC5B was calculated as the percentage of the mucus plug area positive for MUC5AC divided by the percentage of the mucus plug area positive for MUC5B (Supplemental Figure 7C). When the airway size exceeded the capture area of the microscope, the airway was scanned repeatedly to cover the entire airway area, and the measurements of each image were averaged to produce a single value per airway.

Imaging mass cytometry (IMC)

The metal-tagged antibodies used in the study are provided in Supplemental Table 2. We mostly used pre-conjugated antibodies purchased from Fluidigm. However, if pre-conjugated antibodies were not commercially available, carrier-free antibodies (pre-

validated by immunohistochemistry) were labeled to metal tags using the MaxPar® labeling kit (Fluidigm), according to manufacturer's guidelines. An auto-stainer (Leica biosystems- Bond™ Rx) was used for slide deparaffinization and antigen retrieval. Briefly, pre-baked formalin-fixed paraffin-embedded (FFPE) tissue sections were deparaffinized in a Bond™ Dewax solution (Leica biosystems) at 72°C for 5 min. The tissue sections were then hydrated in descending concentrations of ethanol and washed using a Bond™ washing solution (Leica biosystems). The antigen retrieval step was performed in Bond™ epitope retrieval solution 2 (pH 9, Leica biosystems) at 96°C for 30 minutes. Slides were then subsequently rinsed with a Bond™ washing solution for 10 minutes and the later steps were performed manually. Slides were washed with milliQ water for 5 minutes then with 1X Dulbecco's phosphate-buffered saline (DPBS) (ThermoFisher) for 10 minutes. The blocking step was carried out using freshly prepared 3% bovine serum albumin (BSA) (Sigma) for 45 minutes and the antibody cocktail (in 0.5% BSA - DPBS) was incubated overnight at 4°C. After staining, slides were washed twice with 0.2% Triton-X100, followed by two DBPS washes. A cationic nucleic acid intercalator (Cell-ID Intercalator-Ir, Fluidigm) was used to stain the DNA (1:500, 30 minutes) before a last water wash and the air-drying of the slides. Slides were lastly scanned, using a regular scanner (Canon LiDE 210), to get an optical image of the tissue sections and for identification of mucus plugs and non-obstructed airways. Once all the regions of interest (ROIs) have been determined, the cocktail-stained slides were scanned with the Fluidigm Hyperion Plus. The ROI size was adjusted for each airway. The system used a UV laser to ablate the tissue at a 1µm resolution. The metal isotopes were then ionized and detected with a mass cytometer.

The IMC process generated mcd files that contained metal isotype detection information, including their coordinates.

Imaging mass cytometry (IMC) data analysis

The IMC pipeline for data analysis and cell segmentation is illustrated in Supplemental Figure 8.

Image pre-processing

The mcd files were first converted to ome.tiff files using the readimc python package (https://github.com/BodenmillerGroup/readimc). This step created two multi-channel image stacks for each ROI: the full stack and the ilastik stack. The full stack includes all channels that will be later used for image visualization and to assess the expression of cell-specific markers. The ilastik stack includes selected markers necessary for pixel training (DNA1, DNA2, CD45, aSMA, E-Cad, CK5, and Pan-actin).

Cell segmentation

The IMC Segmentation Pipeline was used for cell segmentation (https://github.com/BodenmillerGroup/ImcSegmentationPipeline). Briefly, the ilastik stack is loaded into Ilastik to generate probabilities for nuclei, cytoplasm, and background pixels. The pixel-level probabilities are then loaded on CellProfiler to segment individual cells. The CellProfiler pipeline not only provides cell masks, delineating the outer boundary of all individual cells within an image, but also extracts single cell features such as mean intensity per channel and spatial neighbor information. The accuracy of single

cell segmentation was verified on R using the cytomapper package, that allows IMC image visualization (https://github.com/BodenmillerGroup/cytomapper).

Downstream analysis

The data generated by the IMC Segmentation Pipeline were imported in R using the imcRtools package (https://github.com/BodenmillerGroup/imcRtools) to create a SpatialExperiment object. Sample specific metadata were added to each ROI, such as subject status (asthma, lung disease-free control^{Asthma}, COPD, lung disease-free control^{COPD}) but also airway obstruction status (plugged, unplugged). Prior to analysis, the expression counts were transformed using an inverse hyperbolic sine function (co-factor 1) to avoid an analysis bias that could come from the typically low numbers observed in the IMC data. Before further analysis, cell-level quality was evaluated. Cells had an average area of 132 pixels and cells smaller than 5 pixels (74 cells) and higher than 4000 pixels (6 cells) were excluded from the study. The signal intensity and signal-to-noise ratio (SNR) at the single cell level were evaluated to check the signal quality of each antibody. CD4, IL-3RB and BCL-XL presented the lower signal intensity and SNR (Supplemental Figure 9A). In addition, BCL-2 immunostaining was not consistent between samples. The 4 markers were thus not considered in the future analysis. The multidimensional data were visualized in low dimensional space using a Uniform Manifold Approximation and Projection (UMAP). We observed that cells clustered according to the subject status rather than phenotypic similarities (Supplemental Figure 9B). To address this issue, we used the Seurat package to correct the batch effect by aligning and integrating cells from the different samples (2). Briefly, a Seurat object was created from

the SpatialExperiment object. We used the FindIntegrationAnchors function with a k.anchor parameter of 20, followed by the IntegrateData function. The cell coordinates generated with the PCA dimensionality reduction were then transferred to the SpatialExperiment object. The cells exhibited a more homogeneous distribution in the UMAP plot after the Seurat correction (Supplemental Figure 9C).

To identify cell classes and cell types, the PhenoGraph clustering approach was used *(3)*. We set the nearest neighbors at 25, the random seed at 2, and other parameters were set as default. The different clusters generated were then annotated regarding the expression of the different markers (Supplemental Table 3 and Figure 2D). A subset of 29 markers were used to identify 23 different cell types (Figure 2D). The markers excluded for cell type identification where either DNA markers (DNA1, DNA2, Histone H3), a structure marker (pan-actin) or markers excluded from the quality check analysis (BCL-2, BCL-XL, CD4 and IL-3RB, Supplemental Figure 9A). Five cell classes were identified: unassigned (cells negative for all markers), smooth muscle cells (α-Smooth-muscle actin positive cells), epithelial cells (E-Cadherin positive cells), endothelial cells (CD31 positive cells) and immune cells (cells positive for immune cells markers : CD45, CD3, CD20, CD8, CD57, CD11b, CD11c, EPX, ELA, Mast cell tryptase, mast cell chymase, major basic protein, CD68, CD14, HLA-DR, IL-5RA, CD163, IL-33R, IL-4, CD303 and CD123) (Figure 2B). The five cell classes were then subdivided into 23 cell types (Supplemental Table 3 and Figure 2D).

To spatially categorize cells within the airway (airway epithelium, airway lumen or airway wall), we drawn the airway lumen and airway epithelium of each region of interest (ROI) scanned with the Hyperion. In practice, full stacks generated during the pre-processing phase were opened with ImageJ and structural markers and DNA staining were used to delineate airway structures. The coordinates of lumen and epithelium shapes were then saved and imported in R. For each cell included in the different ROIs, we compared the coordinates of the cell with those of the lumen and epithelium shapes. If a cell's coordinates fell within the predefined limits of the airway lumen or epithelium, the cell was assigned to that specific airway location. Cells whose coordinates did not fall within these zones were assigned to the airway wall.

Finally, taking into account the spatial location of each cell, we generated counts for each cell type and performed analyses to assess cell type abundance in the different groups (asthma plugged airways, asthma unplugged airways, disease-free control^{Asthma} airways, COPD plugged airways, COPD unplugged airways and disease-free control^{COPD} airways). When the airway size exceeded the Hyperion system's capture zone, the airway was scanned using multiple ROIs and cell counts from each individual ROI were summed. Given the variability in airway size, we normalized cell counts by basement membrane length for cells located in the airway wall and the airway epithelium, and by lumen area for cells located in the airway lumen. All images showing cell segmentation and cell class/type identification were generated with the plotCells function of the cytomapper package.

Immunostaining visualization and quantification

Extracellular staining for mucins (MUC5AC and MUC5B) and Histone H3 could not be assessed using single-cell segmentation. Instead, the plotPixel function from the cytomapper package was used to visualize mucin immunostaining and confirm their presence in the lumen of plugged airways. The visualization of the Histone H3 immunostaining was also done with the plotPixel function, enhanced by the presence of single-cell segmentation to distinctly illustrate the extracellular staining patterns. The quantification of the Histone H3 immunostaining in the airway lumen of plugged airways was done with ImageJ. In practice, the airway lumen was traced on each image and the mean intensity signal of the Histone H3 immunostaining was calculated using the Otsu thresholding method (4).

Human airway epithelial cells and eosinophils co-cultures

Human airway epithelial cell cultures

Human airway (trachea) epithelial cells (HAECs) were obtained from donor lungs through the California Transplant Donor Network. Cells were expanded and cultured at air-liquid interface (ALI) following the protocol adapted from Everman *et al* (5). Initially, HAECs were cultured in medium 1, consisting of 47.5% Dulbecco's modified eagle medium (DMEM)/F12 GlutaMAX (Gibco), 47.5% F12 GlutaMAX (Gibco), 5% heat-inactivated fetal bovine serum (FBS) (Sigma-Aldrich), 24 µg/mL adenine (Sigma-Aldrich), 10 ng/mL human epidermal growth factor (Millipore Sigma), 400 ng/mL hydrocortisone (Sigma-Aldrich), 5 µg/mL insulin (Sigma-Aldrich), 8.6 ng/mL cholera toxin (Sigma-Aldrich), and rho-associated protein kinase (ROCK) inhibitor (10 µM, Selleckchem). This first step was

done in Petri dishes precoated with rat tail collagen (Corning). Upon reaching 80% confluence, the cells were harvested using Trypsin 0.25% (Gibco) and seeded onto 12 mm transwell inserts (Corning) precoated with human placental collagen (Sigma). For this stage, cells were cultured in medium 2 containing 50% DMEM GlutaMAX (Gibco), 50% bronchial epithelial cells basal medium (BEBM) (Lonza) with BEBM supplements (Lonza), 50 µg/mL BSA (Sigma-Aldrich), 0.005% Ethanolamine (Sigma-Aldrich), 0.3 µM $MgCl_2$, 0.4µM $MgSO_4$, 1 µM $CaCl_2$ (Sigma-Aldrich), 30 ng/mL retinoic acid (Sigma-Aldrich), and 10 ng/mL human growth factor (Gibco). When the cells achieved 100% confluence, the media was removed from the apical chamber, and cells continued to be cultured in ALI-maintenance medium (PneumaCult ALI complete basal medium, 400 ng/mL heparin, 1 µM hydrocortisone, Stemcell) for 21 days. Antibiotics (50 U/mL Penicillin-streptomycin, 2.5 µg/mL amphotericin B and 50 µg/mL Gentamicin, Gibco) were used throughout. An asthma-like mucus layer was induced by stimulating the cells with interleukin (IL)-13 (10 ng/mL, Peprotech) every other day from day 14 to day 21. To generate mucus-free ALI cultures, the apical chamber was washed with 300 µL of 10 mM dithiothreitol (DTT, Bio-rad) for 10 minutes at 37°C and 5% CO_2. This was followed by two consecutive 10-minutes washes with PBS. This step was done 30 minutes before eosinophil deposition.

Apical secretion recovery and centrifugation

After 21 days of culture at ALI, the apical chambers of IL-13-treated HAECs were washed with 300 µL of PBS for 10 minutes at 37°C and 5% CO_2. For periodate treatment, a 6 mM solution of $NaIO_4$ (Sigma-Aldrich) was used for 2 hours. The high and low molecular weight fractions of apical washes were then separated using Amicon Ultra centrifugal

filters (Millipore Sigma) with a 100,000 Dalton molecular weight cutoff. The eluate was considered the low molecular weight fraction (<100kD). Before recovery, the high molecular weight fraction (>100kD) was washed three times with PBS for buffer exchange.

Eosinophil isolation

Eosinophils were isolated from residual samples from peripheral blood mononuclear cell isolation performed in the context of human clinical studies at the Airway Clinical Research Center, University of California, San Francisco (UCSF). Briefly, the blood was diluted twice in Roswell Park Memorial Institute (RPMI)-1640 medium (Gibco). Thirty milliliters of the diluted blood was layered over 12 mL of Lymphoprep (StemCell), and the sample was centrifuged at 800g for 30 minutes without break. The red pellet was collected and resuspended in 45 mL of 1x Red Blood Cell Lysis Solution (Miltenyi Biotec) for 15 minutes. The sample was centrifuged at 300g for 8 minutes and the supernatant was discarded. The pellet was then washed with 50 mL of buffer (PBS, 5% BSA, 2 mM Ethylenediaminetetraacetic acid [EDTA]) and centrifuged again. Eosinophils were isolated from the lysed human peripheral blood through negative selection using the Eosinophil Isolation Kit, human (Miltenyi Biotec). The magnetic labeling of cells and magnetic separation were performed according to the manufacturer's kit instructions. Eosinophil purity was assessed by differential counts and was higher than 95% for each donor (n=8). Freshly isolated eosinophils were resuspended in PBS at the concentration of 100 000 cells per 30 µL. Subsequently, 100 000 eosinophils were added to the apical chamber of the HAECs in ALI culture and incubated for 48 hours at 37°C and 5% CO_2.

For basal chamber experiments, 100,000 eosinophils were added to 1 mL of ALI-maintenance medium, with or without the presence of a transwell insert containing IL-13-activated HAECs at ALI.

In the case of culturing eosinophils with apical secretion fractions, 100,000 cells were added to 300 µL of PBS containing either the low molecular weight fraction or the high molecular weight fraction of mucus pre-treated or not with $NaIO_4$, anti-CD11b antibody (10 µg/mL, 2LPM19c, Santa Cruz Biotechnology), or mouse isotype control antibody (10 µg/mL, eBioscience).

Eosinophils read-out experiments

After 48 hours of co-culture, the apical chambers of transwell inserts were washed with 300 µL of PBS for 10 minutes at 37°C. Washes from 3 transwell inserts were recovered and pooled together. The washes were centrifuged at 4000 rpm for 4 minutes at 4 °C and only the cell pellet was used for the cell viability and apical washes read-out experiments. For flow cytometry experiments, cells were first stained with an anti-CD45 antibody (1:100, clone 2D1, eBioscience) for 30 minutes on ice. The cells were then washed with PBS and centrifuged at 4000 rpm for 4 minutes at 4°C. Subsequently, cells were stained for 15 minutes at room temperature with AlexaFluor 647-conjugated Annexin V (Thermo Fisher) and Sytox Green (Thermo Fisher) according to the manufacturer's instructions. Data acquisition was carried out using a Fortessa FACS (BD Biosciences), and data analysis was performed on FlowJo after exclusion of debris and doublets. In case of basal chamber experiments or apical secretion fraction experiments, eosinophils were

recovered, centrifuged and underwent the same treatment as the cells incubated in the apical chamber of HAECs at ALI.

For cytospin of apical washes, 30 000 cells were resuspended in PFA 4% (Electron Microscopy Sciences) and seeded on slides with a cytocentrifuge (Thermo Scientific). Cells were permeabilized with triton X-100 0.1% buffer (Sigma-Aldrich) and a PBS buffer containing 10% goat serum (Gibco) and 0.05 % Tween 20 (Sigma-Aldrich) was used to prevent nonspecific antibody binding. Slides were stained overnight at 4°C with a mouse monoclonal anti-MUC5AC antibody (1:300, 45M1, ThermoFisher) and a rabbit polyclonal anti-EPX antibody (1:200, Diagnostics Development). The following day, Cy-3 goat anti-mouse and Cy-5 goat anti-rabbit antibodies (1:300, Jackson Immunoresearch) were applied. Nuclei were stained with Sytox Green (1:10,000), and images were acquired with the Olympus Fluoview 10i laser scanning confocal microscope at 60x magnification (oil immersion objective).

For immunostaining of whole-mounts, no wash was necessary. Cell culture transwell inserts were directly fixed with Carnoy's solution for 30 minutes, followed by 2 washes with methanol and ethanol. Membranes were excised from the inserts, then rehydrated sequentially through a graduated ethanol series and water. Antigen retrieval involved heating the membranes in a citrate buffer (Vector Laboratories) at 99°C for 20 minutes. After cooling for one hour, the membranes underwent cell permeabilization for 15 minutes using 0.1% Triton X-100 buffer (Sigma-Aldrich). To block non-specific binding, membranes were pre-treated with a blocking buffer containing 10% goat serum (Gibco)

and 0.05% Tween 20 (Sigma-Aldrich). Membranes were stained overnight with a mouse monoclonal anti-MUC5AC antibody (1:300, clone 45M1, Invitrogen) and a rabbit polyclonal anti-EPX antibody (1:200, Diagnostics Development). The following day, Cy-3 goat anti-mouse and Cy-5 goat anti-rabbit antibodies (Jackson Immunoresearch) were added at the concentration of 1:300. Nuclei were stained with Sytox Green (1:10,000), and Z-stacks images were acquired with the Olympus Fluoview 10i laser scanning confocal microscope at 60x magnification (oil immersion objective). The three-dimensional rendering and 3D volume reconstruction of mucus, eosinophils and DNA were performed in Imaris 10.2.

Supplemental Figures and Legends

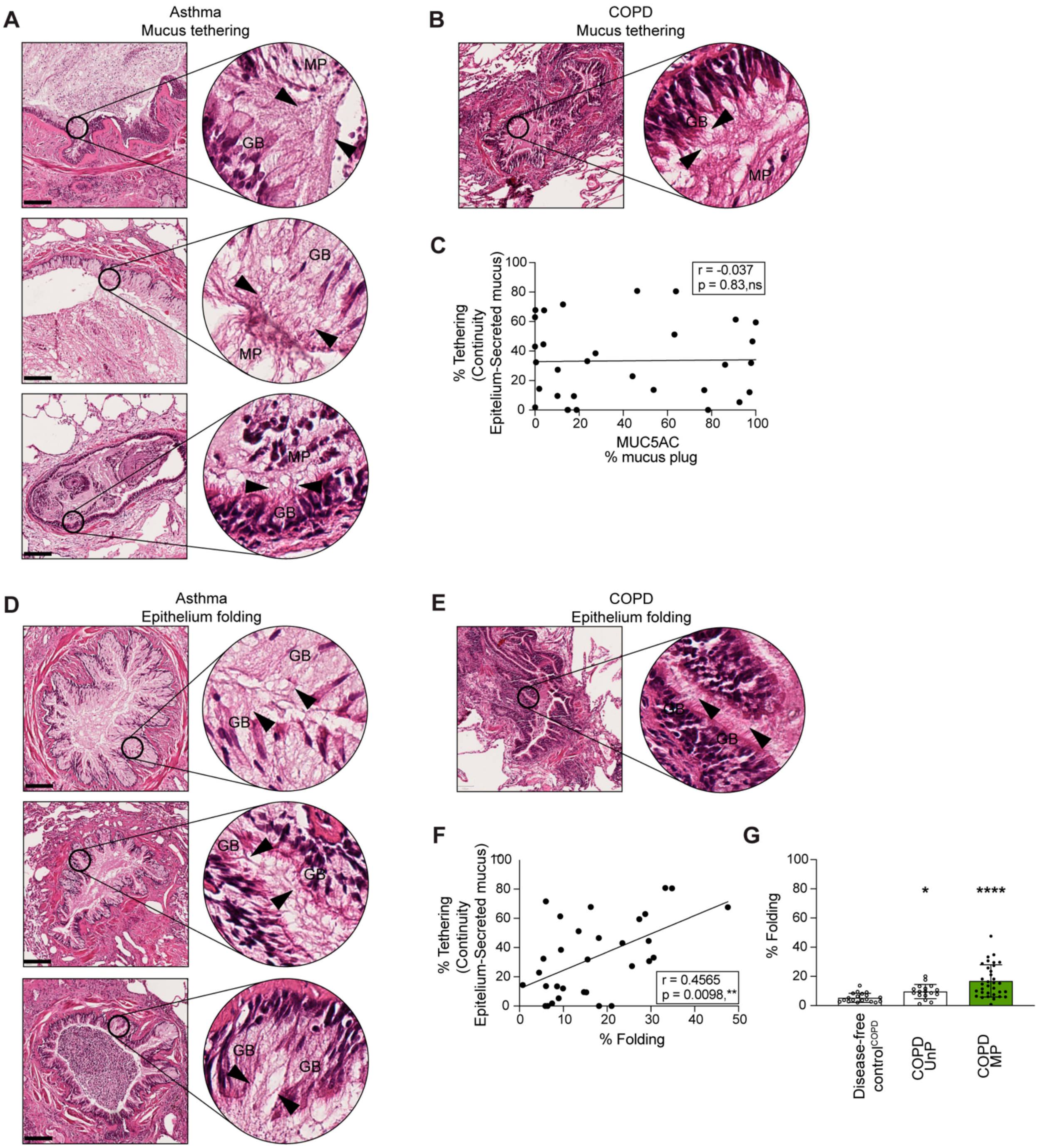

Supplemental Figure 1. Extent of mucus plug tethering to the epithelium and mucosal fold formation in asthma and COPD mucus plugs.

(A) Mucus strands (black arrowheads) connect asthma mucus plugs (MP) to the surface of goblet cells (GB) (subject IDs: 7188, 7232 and 7239). **(B)** Mucus strands (black arrowheads) connect the COPD mucus plug (MP) to the surface of goblet cells (GB) (subject ID: 7336). **(C)** The extent of mucus tethering to the epithelium is not significantly correlated with the intensity of MUC5AC immunostaining within the same mucus plug (n=31) in COPD (Spearman's correlation). **(D)** Mucosal folds in mucus plugged airways in asthma are rich in goblet cells (GB) and mucus is tethered to goblet cells at multiple points along the folds (black arrowheads) (subject IDs: 7232, 7187 and 7298). **(E)** Mucosal folds in mucus plugged airways in COPD are rich in goblet cells (GB) and mucus is tethered to goblet cells at multiple points along the folds (black arrowheads) (subject ID: 6965). **(F)** Mucus plug tethering % correlates with mucosal folding % in mucus plugs in COPD (n=31) (Spearman's correlation). **(G)** Mucosal folding % is higher in mucus plugged airways in COPD (COPD MP) than in unplugged airways in COPD (COPD UnP), or in lung disease-free control^{COPD} airways. *Significantly different from disease-free control^{COPD}, $P<0.05$, ****Significantly different from disease-free control^{COPD}, $P<0.0001$ (Kruskal-Wallis test with Dunn's correction). Scale bars = 200 μm.

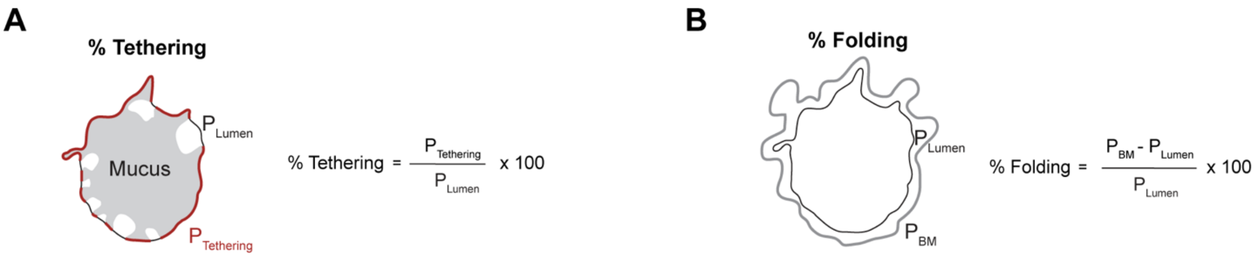

Supplemental Figure 2. Method for quantifying epithelial mucus tethering and mucosal folding.

(A) The percentage of mucus tethering to the epithelium was defined as the percentage of lumen perimeter that present continuity between secreted mucus and epithelial cells (tethering perimeter). **(B)** The percentage of folding was defined as the percentage of the difference between the basement membrane perimeter and the lumen perimeter, divided by the basement membrane perimeter.

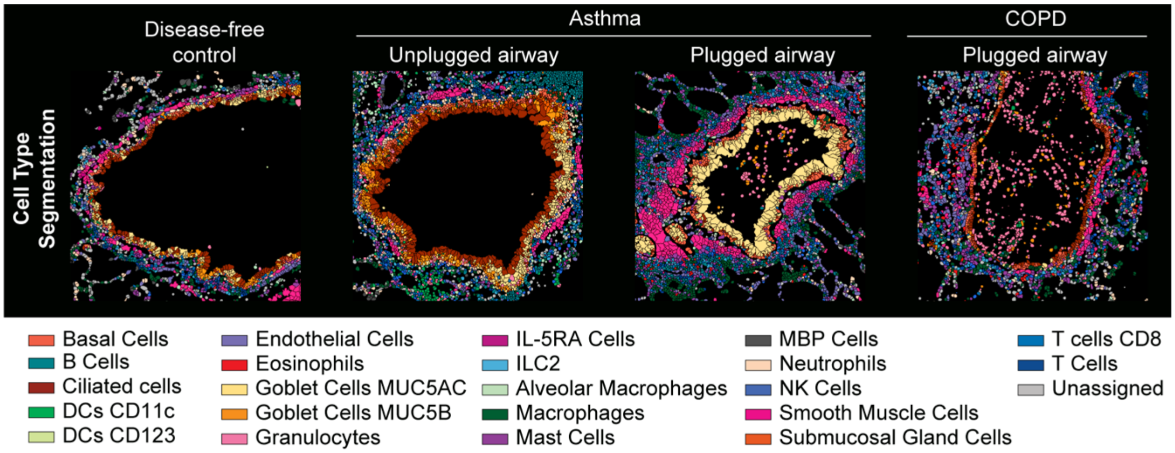

Supplemental Figure 3. Cell type segmentation images.

Representative images of cell type distribution after cell segmentation in a lung disease-free control airway (subject ID: 7272), an asthma unplugged airway (subject ID: 7239), an asthma mucus plugged airway (subject ID: 7188), and a COPD mucus plugged airway (subject ID: 6971).

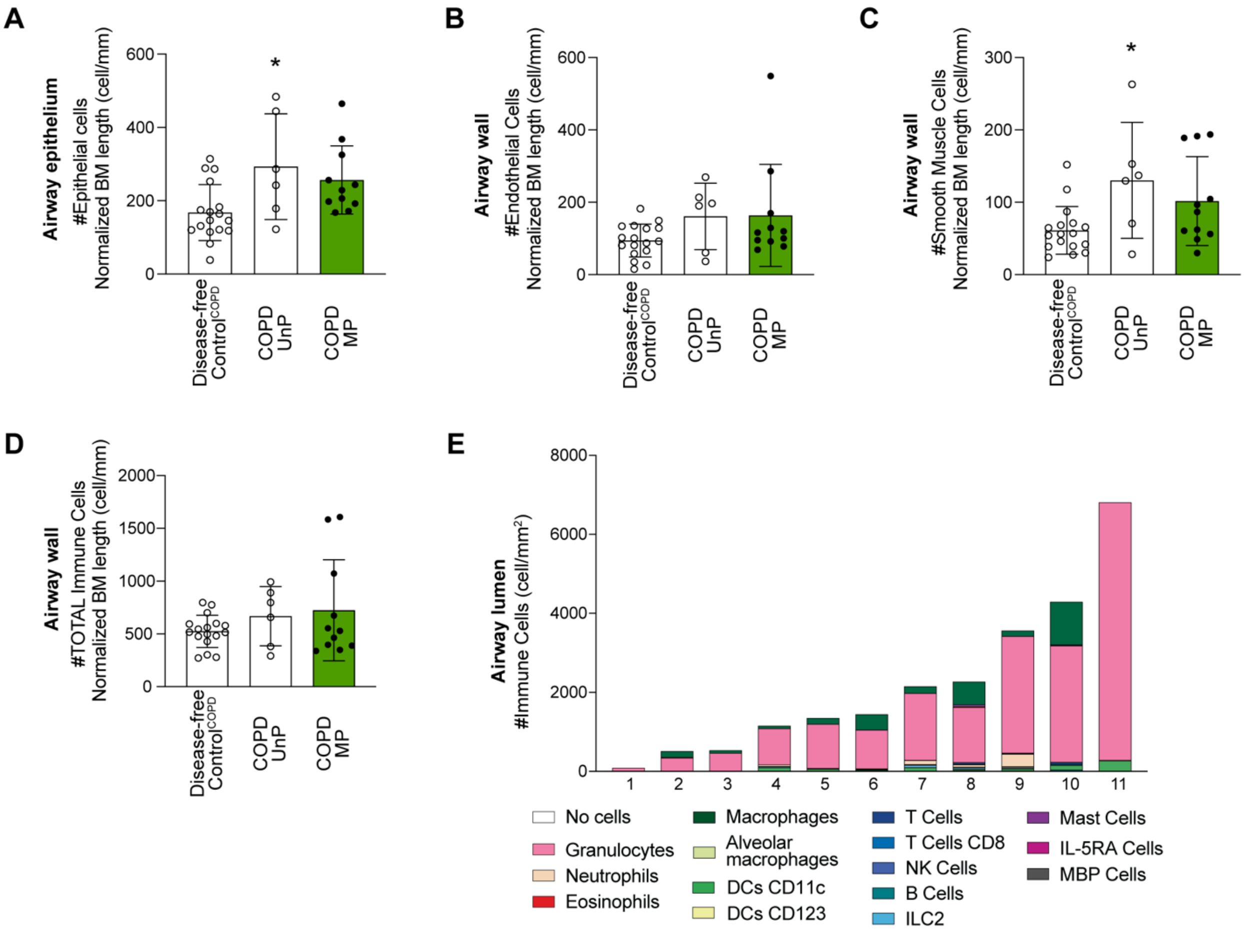

Supplemental Figure 4. Cell composition of airway wall, airway epithelium and airway lumen in COPD mucus unplugged and plugged airways and control airways. **(A)** Epithelial cell numbers in the airway epithelium are similar in COPD mucus plugged airways and disease-free control^{COPD} airways. *Significantly different from disease-free control^{COPD} airways, P<0.05 (ordinary one-way ANOVA with Tukey's correction). **(B)** Endothelial cell numbers in airway wall are similar in COPD mucus plugged airways and disease-free control^{COPD} airways. **(C)** Smooth muscle cell numbers in airway wall are similar in COPD mucus plugged airways and disease-free control^{COPD} airways. *Significantly different from disease-free control^{COPD} airways, P<0.05 (ordinary one-way ANOVA with Tukey's correction). **(D)** Immune cell numbers in airway wall are similar in

COPD mucus plugged airways and disease-free control[COPD] airways. **(E)** Diversity and prevalence of immune cell types infiltrating the lumen of COPD mucus plugs, with granulocytes identified as the predominant immune cell type.

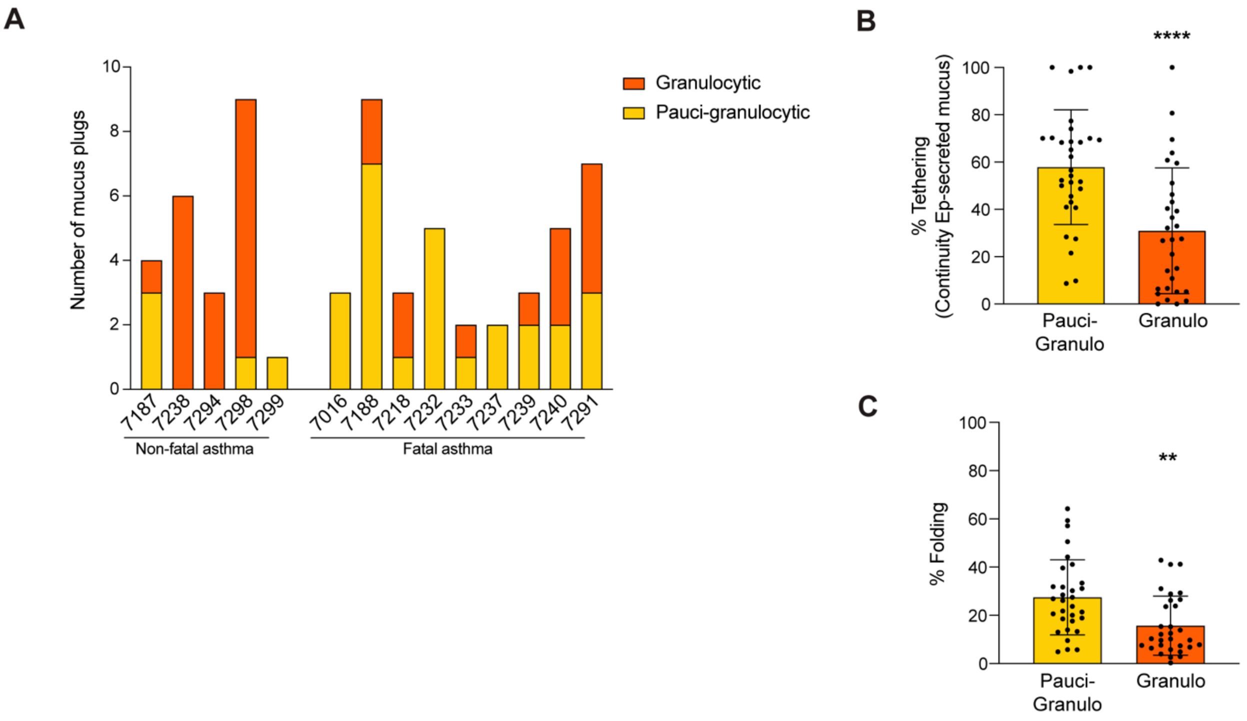

Supplemental Figure 5. The frequency of pauci-granulocytic mucus plugs and granulocytic mucus plugs differs in fatal and non-fatal asthma cases.

(A) Segregation of mucus plug subtypes by subject. **(B)** Mucus plug tethering % in pauci-granulocytic mucus plugs is higher than in granulocytic mucus plugs. ****Significantly different from pauci-granulocytic plugs, p<0.0001 (Mann-Whitney test). **(C)** Mucosal folding % in pauci-granulocytic mucus plugs is higher than in granulocytic mucus plugs. **Significantly different from pauci-granulocytic plugs, p<0.01 (Mann-Whitney test).

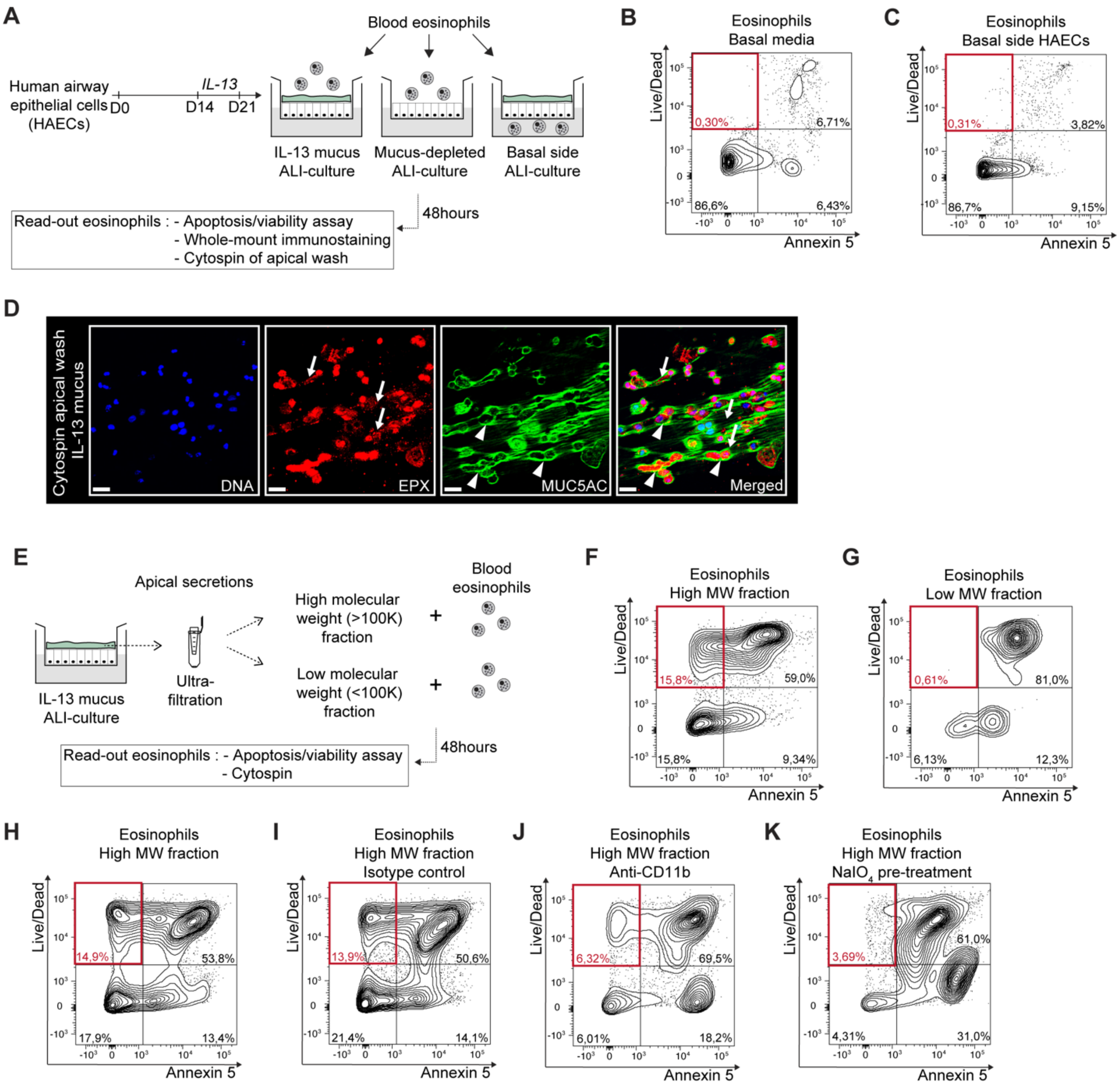

Supplemental Figure 6. Mucus secreted by IL-13 activated airway epithelial cells causes eosinophil cytolytic degranulation. (A) Schematic illustrating the workflow for co-culture of human airway epithelial cells (HAECs) at air-liquid interface (ALI) and human blood eosinophils. **(B)** Non-apoptotic cell death (dead$^+$/annexin5$^-$) does not occur when eosinophils are cultured in basal cell medium. **(C)** Non-apoptotic cell death (dead$^+$/annexin5$^-$) does not occur when eosinophils are cultured in conditioned media in

the basolateral chamber of HAECs cultures. **(D)** Representative immunostaining of apical washes of co-cultures between HAECs and eosinophils. EPX (red), MUC5AC (green) and nuclei (blue). The white arrows show eosinophil degranulation, and the white arrowheads show eosinophils coated with MUC5AC. Scale bar= 20 µm. **(E)** Schematic illustrating the workflow for separating high molecular weight (MW) and low MW fractions of HAECs apical mucus by ultrafiltration. The fractions are then incubated with human blood eosinophils for 48 hours. **(F)** The apoptosis/viability assay shows that 15.8% of eosinophils underwent non-apoptotic death (dead$^+$/annexin5$^-$ cells) when incubated with epithelial high MW mucus. **(G)** Only 0.61% of eosinophils underwent non-apoptotic death (dead$^+$/annexin5$^-$ cells) when incubated with epithelial low MW fraction of apical secretions. **(H)** The apoptosis/viability assay shows that 14.9% of eosinophils underwent non-apoptotic death (dead$^+$/annexin5$^-$ cells) when incubated with epithelial high MW mucus. **(I)** The apoptosis/viability assay shows that 13.9% of eosinophils underwent non-apoptotic death (dead$^+$/annexin5$^-$ cells) when incubated with isotype control antibodies and epithelial high MW mucus. **(J)** The apoptosis/viability assay shows that only 6.32% of eosinophils underwent non-apoptotic death (dead$^+$/annexin5$^-$ cells) when they are pre-treated with anti-CD11b antibodies and incubated with epithelial high MW mucus. **(K)** The apoptosis/viability assay shows that only 3.69% of eosinophils underwent non-apoptotic death (dead$^+$/annexin5$^-$ cells) when incubated with epithelial high MW mucus pre-treated with sodium periodate ($NaIO_4$).

.

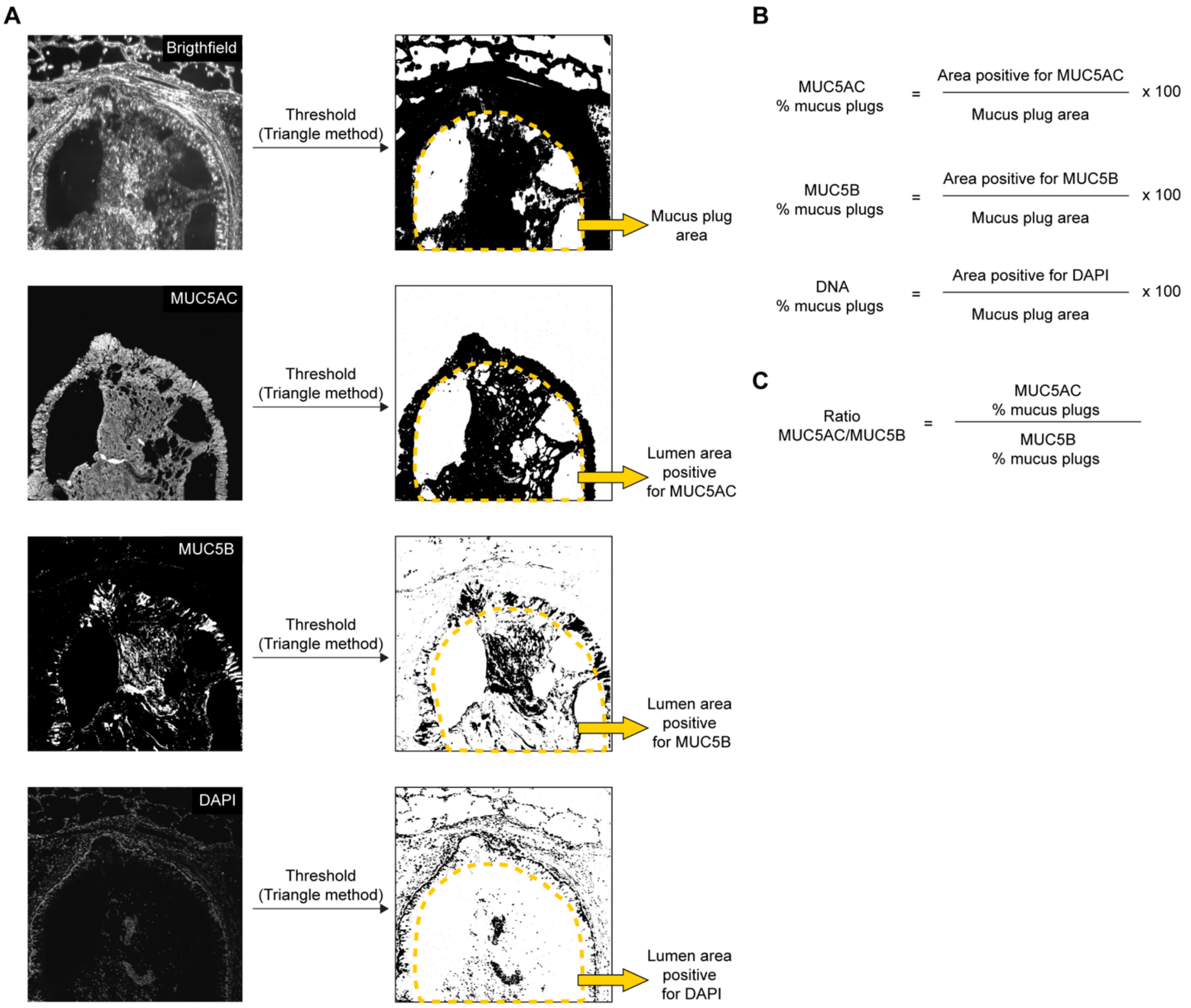

Supplemental Figure 7. Method for quantifying fluorescent immunostainings.

(A) Representative picture of intensity image projection for the brightfield channel or MUC5AC, MUC5B and DNA channels. The triangle algorithm was used as thresholding method and only the black area within the lumen was considered as positive for each staining. **(B)** Method to determine the percentage of mucus plug positive for MUC5AC, MUC5B or DNA. **(C)** Method to determine the MUC5AC to MUC5B ratio.

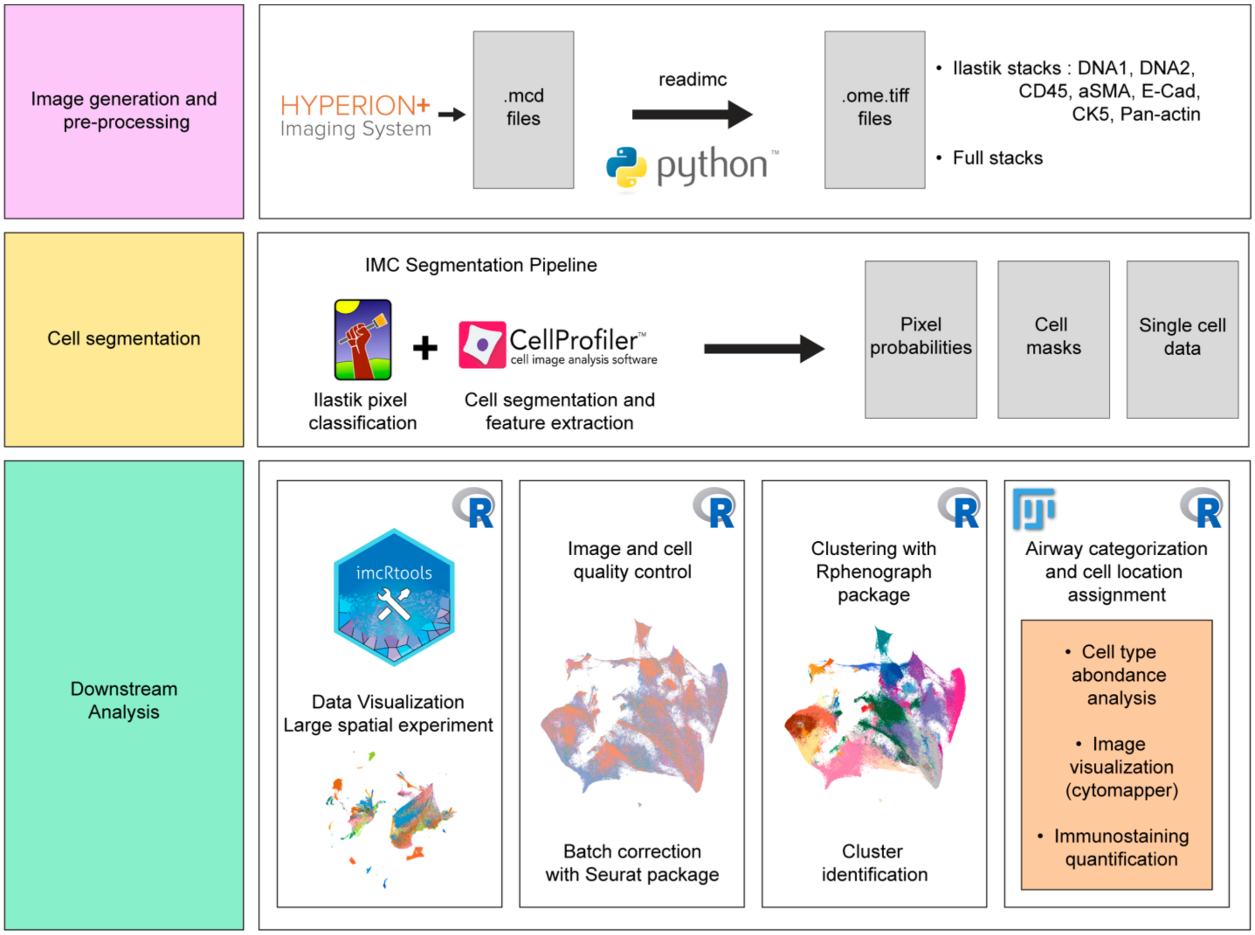

Supplemental Figure 8. Imaging mass cytometry data analysis pipeline.

Images generated with the Hyperion plus were pre-processed with the readimc python package, followed by pixel classification (Ilastik) and cell segmentation (CellProfiler) through the IMC Segmentation pipeline. All downstream analyses were performed in R using the imcRtools package. The final outputs are cell type abondance analysis, image visualization and immunostaining quantification.

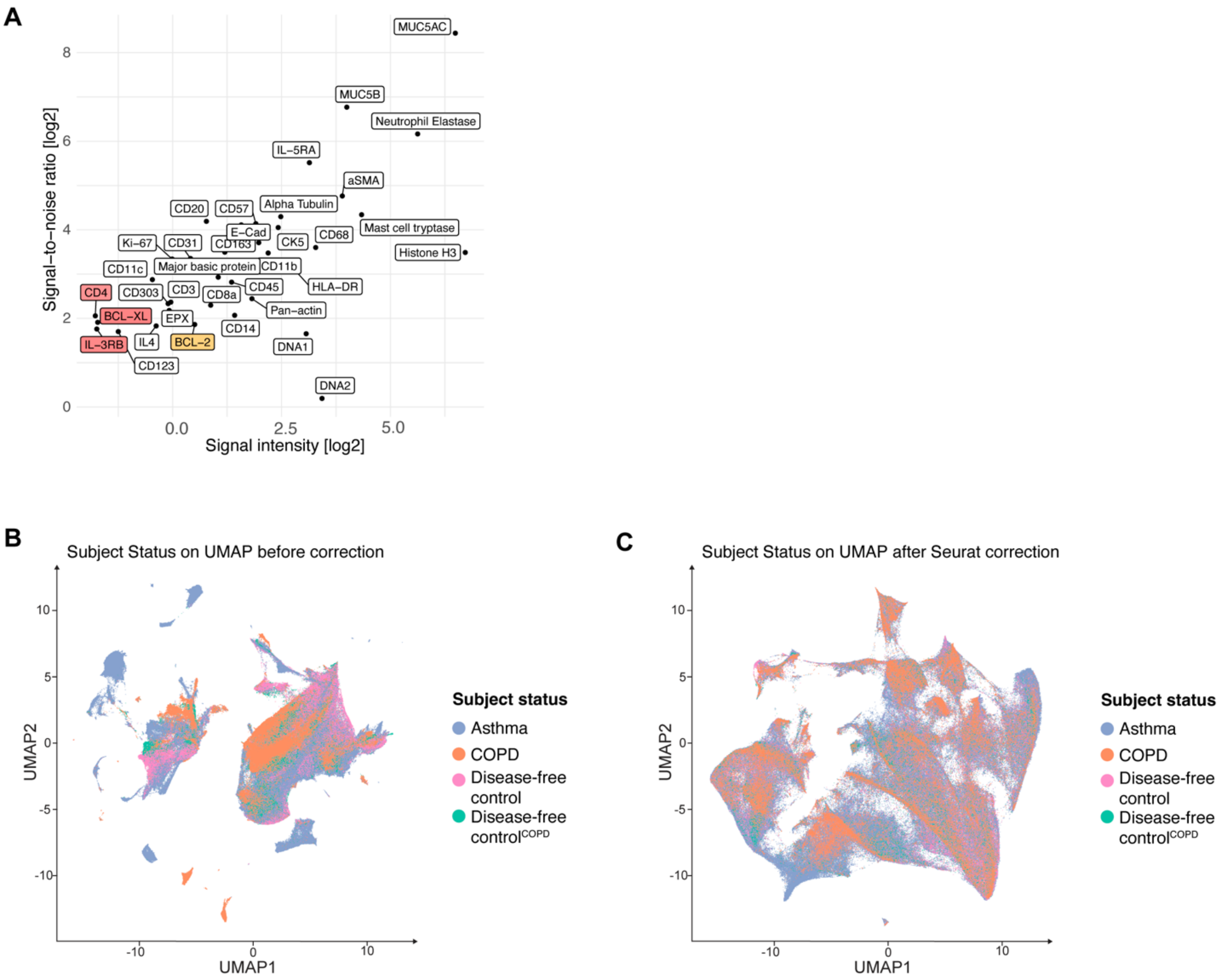

Supplemental Figure 9. Imaging mass cytometry cell-level quality evaluation.

(A) Log2 of the signal intensity of signal to noise ratio for each marker included in the IMC panel. CD4, BCL-XL and IL-3RB (red) presented the lower signal intensities and were excluded from the analysis. In addition, BCL-2 (yellow) did not show consistent staining across samples and was also excluded from the analysis. **(B)** Uniform Manifold Approximation and Projection (UMAP) plot showing cell clustering according to the subject status, before correction. **(C)** Uniform Manifold Approximation and Projection (UMAP) plot showing uniform subject status distribution after Seurat correction for batch effect.

Supplemental Tables

Supplemental Table 1. Details of human airways included in the different analyses.

Subject ID	Status	#Airway	Airway size	*Immuno-fluorescence*	*Tethering*	*Folding*	*Imaging mass cytometry*
7187	Asthma (Non-fatal)	MP1	Small airway	X	X	X	X
		MP2	Small airway	X	X	X	X
		MP3	Small airway	X	X	X	
		MP4	Small airway	X	X	X	
		UnP1	Small airway			X	X
		UnP2	Small Airway				X
		UnP3	Small airway			X	X
		UnP4	Small airway			X	X
		UnP5	Small airway			X	
7238	Asthma (Non-fatal)	MP1	Large airway	X	X	X	X
		MP1	Large airway	X	X	X	
		MP3	Small airway	X	X	X	X
		MP4	Small airway	X	X	X	
		MP5	Small airway	X	X	X	
		MP6	Small airway	X	X	X	
		UnP1	Small airway			X	X
		UnP2	Small airway			X	X
7294	Asthma (Non-fatal)	MP1	Large airway	X	X	X	X
		MP2	Small airway	X	X	X	
		MP3	Small airway	X	X	X	
		UnP1	Large airway			X	
		UnP2	Small airway			X	
		UnP3	Small airway				X
7298	Asthma (Non-fatal)	MP1	Large airway	X	X	X	X
		MP2	Large airway	X	X	X	X
		MP3	Small airway	X	X	X	X
		MP4	Small airway	X	X	X	
		MP5	Small airway	X	X	X	
		MP6	Small airway	X	X	X	
		MP7	Small airway	X	X	X	
		MP8	Small airway	X	X	X	
		MP9	Small airway	X	X	X	
		UnP1	Small airway			X	X
7299	Asthma (Non-fatal)	MP1	Small airway	X	X	X	X
		MP2	Small airway				X
		UnP1	Small airway			X	X
		UnP2	Small airway			X	X
7016	Asthma (Fatal Asthma)	MP1	Large airway	X	X	X	
		MP2	Small airway	X	X	X	X
		MP3	Small airway	X	X	X	
		UnP1	Small airway			X	X
		UnP2	Small airway			X	
		UnP3	Small airway			X	
		UnP4	Small airway			X	
		UnP5	Small airway			X	
		UnP6	Small airway			X	

7188	Asthma (Fatal Asthma)	MP1	Large airway	X	X	X	
		MP2	Large airway	X	X	X	
		MP3	Large airway	X	X	X	
		MP4	Large airway	X	X	X	X
		MP5	Small airway	X	X	X	
		MP6	Small airway	X	X	X	
		MP7	Small airway	X	X	X	X
		MP8	Small airway	X	X	X	X
		MP9	Small airway	X	X	X	
		UnP1	Large airway			X	X
		UnP2	Small airway			X	
		UnP3	Small airway			X	
		UnP4	Small airway			X	X
		UnP5	Small Airway				X
		UnP6	Small Airway				X
		UnP7	Small Airway				X
		UnP8	Small airway			X	
		UnP9	Small airway			X	
7218	Asthma (Fatal Asthma)	MP1	Small airway	X	X	X	
		MP2	Small airway	X	X	X	
		MP3	Small airway	X	X	X	
		UnP1	Small airway			X	
		UnP2	Small airway			X	
		UnP3	Small airway			X	
7232	Asthma (Fatal Asthma)	MP1	Small airway	X	X	X	
		MP2	Small airway	X	X	X	X
		MP3	Small airway	X	X	X	X
		MP4	Small airway	X	X	X	X
		MP5	Small airway	X	X	X	X
		UnP1	Small airway			X	X
		UnP2	Small airway			X	X
7233	Asthma (Fatal Asthma)	MP1	Small airway	X	X	X	X
		MP2	Small airway	X	X	X	X
		UnP1	Small airway			X	X
		UnP2	Small airway			X	X
		UnP3	Small airway			X	
7237	Asthma (Fatal Asthma)	MP1	Large airway	X	X	X	
		MP2	Large Airway				X
		MP3	Large Airway				X
		MP4	Small Airway				X
		MP5	Small Airway				X
		MP6	Small Airway				X
		MP7	Small Airway				X
		MP8	Small airway	X	X	X	
		UnP1	Small airway			X	
		UnP2	Small airway			X	
		UnP3	Small airway			X	
		UnP4	Small airway			X	
		UnP5	Small Airway				X
7239	Asthma (Fatal Asthma)	MP1	Large airway	X	X	X	
		MP2	Small airway	X	X	X	X
		MP3	Small airway	X	X	X	
		UnP1	Small airway			X	X
		UnP2	Small airway			X	X
		UnP3	Small airway			X	

7240	Asthma (Fatal Asthma)	MP1	Large airway	X	X	X	
		MP2	Small airway	X		X	
		MP3	Small airway	X	X	X	
		MP4	Small airway	X	X	X	
		MP5	Small airway	X	X	X	
		UnP1	Small airway			X	
		UnP2	Small airway			X	
		UnP3	Small airway			X	
7291	Asthma (Fatal Asthma)	MP1	Large airway	X	X	X	X
		MP2	Large airway	X	X	X	
		MP3	Small airway	X	X	X	
		MP4	Small airway	X	X	X	X
		MP5	Small airway	X	X	X	X
		MP6	Small airway	X	X	X	X
		MP7	Small airway	X	X	X	X
7018	Disease-free control	C1	Small airway			X	
		C2	Small airway			X	
		C3	Small airway			X	
		C4	Small airway			X	X
		C5	Small airway			X	X
		C6	Small airway				X
		C7	Small Airway				X
7219	Disease-free control	C1	Large Airway			X	
		C2	Large Airway			X	
		C3	Small Airway				X
		C4	Small Airway				X
		C5	Small Airway				X
		C6	Small Airway				X
		C7	Small Airway				X
7220	Disease-free control	C1	Large Airway			X	
		C2	Large Airway			X	
		C3	Large Airway				X
		C4	Large Airway				X
		C5	Small Airway				X
		C6	Small Airway				X
		C7	Small Airway				X
7234	Disease-free control	C1	Large Airway			X	
		C2	Large Airway			X	X
		C3	Small airway			X	
		C4	Small airway			X	
		C5	Small airway			X	X
		C6	Small airway			X	
		C7	Small airway				X
7259	Disease-free control	C1	Large Airway			X	
		C2	Large Airway				X
		C3	Large Airway			X	X
		C4	Small Airway			X	X
7272	Non-diseased control	C1	Small airway			X	X
		C2	Small Airway			X	X
		C3	Small Airway			X	
		C4	Small airway			X	
7280	Disease-free control	C1	Small airway			X	X
		C2	Small airway			X	X
		C3	Small airway				X
7293	Disease-free	C1	Large Airway			X	X

	control	C2	Large Airway				X
		C3	Small airway			X	
		C4	Small airway			X	X
		C5	Small Airway			X	
1588	COPD (GOLD3)	MP1	Small Airway	X	X	X	
		MP2	Small Airway	X	X	X	
		UnP1	Small airway			X	
1888	COPD (GOLD3)	MP1	Small Airway	X	X	X	
		MP2	Small Airway	X	X	X	
3142	COPD (GOLD3)	MP1	Small Airway	X	X	X	
		MP2	Small Airway	X	X	X	
		MP3	Small Airway	X	X	X	
		UnP1	Small airway			X	
6965	COPD (GOLD4)	MP1	Large airway	X	X	X	
		MP2	Small airway	X	X	X	
6967	COPD (GOLD4)	MP1	Large Airway	X	X	X	
		MP2	Small Airway	X	X	X	
		MP3	Small Airway	X	X	X	
		MP4	Small Airway	X	X	X	X
		MP5	Small Airway	X	X	X	
		MP6	Small Airway	X	X	X	X
		MP7	Small Airway	X	X	X	X
		MP8	Small airway				X
		UnP1	Large airway			X	
		UnP2	Large airway			X	
		UnP3	Small airway			X	X
		UnP4	Small airway			X	
6968	COPD (GOLD4)	MP1	Large Airway	X	X	X	X
		MP2	Small Airway				X
		MP3	Small Airway	X	X	X	
		UnP1	Small airway			X	
		UnP2	Small Airway				X
6969	COPD (GOLD4)	MP1	Large airway	X	X	X	
		UnP1	Large Airway				X
		UnP2	Small Airway				X
		UnP3	Small airway			X	
6971	COPD (GOLD4)	MP1	Large airway	X		X	
		MP2	Small airway	X		X	
		MP3	Small Airway	X	X	X	
		MP4	Small Airway	X	X	X	X
		MP5	Small Airway	X	X	X	X
		MP6	Small Airway				X
		UnP1	Small airway			X	
7305	COPD (GOLD4)	MP1	Large airway	X	X	X	
		MP2	Large airway	X	X	X	
		MP3	Large airway	X	X	X	
		UnP1	Large airway			X	
		UnP2	Large airway			X	
		UnP3	Large airway			X	
		UnP4	Large airway			X	
7336	COPD (GOLD4)	MP1	Large airway	X	X	X	X
		MP2	Large airway	X	X	X	
		MP3	Small airway	X	X	X	
		MP4	Small airway	X	X	X	
		MP5	Small airway	X	X	X	

		MP6	Small airway	X	X	X	X
		UnP1	Large airway			X	
		UnP2	Large airway			X	
		UnP3	Small airway			X	
		UnP4	Small airway			X	X
		UnP5	Small airway				X
5973	Disease-free control[COPD]	C1	Small Airway			X	
		C2	Small Airway			X	
6019	Disease-free control[COPD]	C1	Large Airway				X
		C2	Small Airway			X	
		C3	Small Airway			X	
		C4	Small Airway				X
		C5	Small Airway				X
6982	Disease-free control[COPD]	C1	Large Airway				X
		C2	Large Airway				X
		C3	Small Airway			X	
		C4	Small Airway			X	
		C5	Small Airway			X	
		C6	Small Airway				X
		C7	Small Airway				X
7009	Disease-free control[COPD]	C1	Large Airway			X	
		C2	Small Airway			X	
		C3	Small Airway			X	
		C4	Small Airway			X	
		C5	Small Airway				X
		C6	Small Airway				X
		C7	Small Airway				X
		C8	Small Airway				X
7184	Disease-free control[COPD]	C1	Large Airway			X	
		C2	Large Airway			X	
		C3	Large Airway			X	
		C4	Large Airway				X
		C5	Small Airway			X	
		C6	Small Airway				X
7309	Disease-free control[COPD]	C1	Large Airway			X	X
		C2	Large Airway			X	
		C3	Small Airway			X	
		C4	Small Airway			X	X
		C5	Small Airway				X
		C6	Small Airway				X
		C7	Small Airway			X	

MP = Mucus plug, UnP = Unplugged airway, C= lung disease-free control airway.

Supplemental Table 2. IMC antibody cocktail.

Company	Catalog number	Antigen	Clone	Metal	Reliable staining observed
Fluidigm	3158029D	E-Cadherin	24E10	Gd158	Yes
Fluidigm	3168022D	Ki-67	B56	Er168	Yes
Fluidigm	3146019D	BCL-2	EPR17509	Nd146	No
Fluidigm	3176023D	Histone H3	D1H2	Yb176	Yes
Fluidigm	3175032D	Pan-actin	D18C11	Lu175	Yes
Fluidigm	3151025D	CD31	EPR3094	Eu151	Yes
Fluidigm	3141017D	α -Smooth-muscle actin	1A4	Pr141	Yes
Fluidigm	3152018D	CD45	D9M8I	Sm152	Yes
Fluidigm	3154026D	CD11c	3.9	Sm154	Yes
Fluidigm	3159035D	CD68	KP1	Tb159	Yes
Fluidigm	3174023D	HLA-DR	LN3	Yb174	Yes
Fluidigm	3147021D	CD163/M130	EDHu-1	Sm147	Yes
Fluidigm	3144025D	CD14	EPR3653	Nd144	Yes
Fluidigm	3162034D	CD8a	C8/144B	Dy162	Yes
Fluidigm	3170019D	CD3	polyclonal	Er170	Yes
Fluidigm	3161029D	CD20	H1	Dy161	Yes
Fluidigm	91H035145	CD57	NK/804	Nd145	Yes
Fluidigm	3149028D	CD11b	EPR1344	Sm149	Yes
R&D	MAB91671	Neutrophil Elastase/ELA2	950317	Dy164	Yes
Novus	NBP2-22194	CK5	2C2	Dy163	Yes

Abcam	ab105460	MUC5B	6F10-E4	Nd143	Yes
Abcam	ab209348	Alpha Tubulin (acetyl K40)	EPR16772	Nd148	Yes
Abcam	ab199099	BCL-XL	E18	Tm169	No
R&D	MAB301	CD123/IL3Ra	32703	Yb173	Yes
R&D	AF1376	CD303	polyclonal	Nd142	Yes
R&D	MAB379	CD4	34930	Gd156	No
Abcam	ab300139	IL4	EPR2569-115	Yb172	Yes
Novus	NBP2-32732	MUC5AC	45M1	Er166	Yes
Diagnostics development	EPO pab	Eosinophil peroxidase, EPX	polyclonal	Nd150	Yes
Abcam	ab251603	IL-5RA	CAL40	Eu153	Yes
Abcam	ab284690	IL-3RB	EP1037Y	Gd155	No
Abcam	ab14462	Major basic protein, PRG2	BMK13	Gd160	Yes
Abcam	ab271916	Mast cell tryptase	EPR9522	Ho165	Yes
Abcam	ab233729	Mast cell chymase	EPR13136	Er167	Yes
Abcam	ab291094	ST2 (IL33 receptor)	EPR25294-147	Yb171	Yes
Fluidigm	201192B	DNA1	/	Ir191	Yes
Fluidigm	201192B	DNA2	/	Ir193	Yes

Supplemental Table 3. IMC Cell class and cell type identification.

**Cell class**	**Cell type**	**Markers**
Epithelial Cells	Basal Cells	E-Cadherin$^+$, CK5$^+$
Immune Cells	B cells	CD20$^+$
Epithelial Cells	Ciliated Cells	Alpha Tubulin$^+$, E-Cadherin$^+$
Immune Cells	DCs CD11c	CD11c$^+$
Immune Cells	DCs CD123	CD123$^+$
Endothelial Cells	Endothelial Cells	CD31$^+$
Immune Cells	Eosinophils	EPX$^+$
Epithelial Cells	Goblet Cells MUC5AC	E-Cadherin$^+$, MUC5AC$^+$
Epithelial Cells	Goblet Cells MUC5B	E-Cadherin$^+$, MUC5B$^+$
Immune Cells	Granulocytes	ELA2$^+$, EPX$^+$, CD11b$^+$, IL-5RA$^+$
Immune Cells	IL-5RA Cells	IL-5RA$^+$
Immune Cells	ILC2	CD45$^+$, IL-33R$^+$, IL-4$^+$
Immune Cells	Alveolar macrophages	CD163$^+$
Immune Cells	Macrophages	CD68$^+$, HLA-DR$^+$, CD163$^+$, CD14$^+$
Immune Cells	Mast Cells	Mast cell tryptase$^+$, Mast cell chymase$^+$
Immune Cells	MBP Cells	Major Basic Protein$^+$
Immune Cells	Neutrophils	ELA2$^+$
Immune Cells	NK Cells	CD57$^+$, CD45$^+$
Smooth muscles	Smooth muscles	aSMA$^+$
Epithelial Cells	Submucosal Glands	MUC5B$^+$, E-Cadherin$^+$
Immune Cells	T cells CD8	CD8a$^+$, CD3$^+$, CD45$^+$

Immune Cells	T cells	$CD3^+$, CD45+
Unassigned	Unassigned	Negative for all markers

Supplemental video

Supplemental Video 1: 3D-rendering of a co-culture between human airways epithelial cells at air-liquid interface culture and eosinophils. Laser-scanning confocal microscopy movie showing the three-dimensional architecture of a co-culture between human airways epithelial cells at air-liquid interface culture and eosinophils. MUC5AC is in green, eosinophil peroxidase in red and the nuclei are in blue. The video shows eosinophil degranulation in the mucus layer.

Supplemental references

1. Zack GW, Rogers WE, Latt SA. Automatic measurement of sister chromatid exchange frequency. *J Histochem Cytochem*. 1977;25(7):741–753.
2. Stuart T, et al. Comprehensive Integration of Single-Cell Data. *Cell*. 2019;177(7):1888-1902.e21.
3. Levine JH, et al. Data-Driven Phenotypic Dissection of AML Reveals Progenitor-like Cells that Correlate with Prognosis. *Cell*. 2015;162(1):184–197.
4. Otsu N. Threshold Selection Method from Gray-Level Histograms. *IEEE Trans Syst Man Cybern*. 1979;SMC-9(1):62–66.
5. Everman JL, Rios C, Seibold MA. Utilization of Air-Liquid Interface Cultures as an In Vitro Model to Assess Primary Airway Epithelial Cell Responses to the Type 2 Cytokine Interleukin-13. *Methods Mol Biol*. 2018;1799:419–432.
